# Teaching Sexual Orientation and Gender Identity in Pediatric Clinical Settings: A Training Workshop for Faculty and Residents

**DOI:** 10.15766/mep_2374-8265.11137

**Published:** 2021-04-05

**Authors:** Caroline R. Paul, Adam D. Wolfe, Marina Catallozzi, Thanakorn Jirasevijinda, Eric Kutscher, Brian Lurie

**Affiliations:** 1 Associate Professor, Department of Pediatrics, University of Wisconsin School of Medicine and Public Health; 2 Assistant Professor, Assistant Dean of Education, and Associate Residency Program Director, Department of Pediatrics, Baylor College of Medicine at The Children's Hospital of San Antonio; 3 Associate Professor, Vice-Chair of Education, and Director of Pediatric Medical Education, Department of Pediatrics, Columbia University Irving Medical Center; Director of the General Public Health Program, Coleader of the Sexuality Sexual and Reproductive Health Certificate, Heilbrunn Department of Population and Family Health, Columbia University Irving Medical Center; 4 Associate Professor, Director of Pediatric Undergraduate Medical Education, Department of Pediatrics, Weill Cornell Medical College; 5 Resident Physician, Department of Internal Medicine-Primary Care, New York University Langone Health, Bellevue Hospital Center; 6 Associate Professor, Director of General Outpatient Pediatric Division, and Assistant Director of Pediatric Residency Program, Department of Pediatrics, Levine Children's Hospital—Atrium Health; Education Task Force Lead for LGBTQA+ Learning Community, Association of Pediatric Program Directors

**Keywords:** Faculty Development, Sexual Orientation, Gender Identity, Pediatrics, LGBTQ+, Resident-as-Teacher, Clinical Teaching/Bedside Teaching, Diversity, Inclusion, Health Equity

## Abstract

**Introduction:**

Health disparities for the lesbian, gay, bisexual, transgender, queer, intersex, asexual, all other genders, sexes, and sexualities (LGBTQIA+) population are striking. Yet, deliberate efforts to integrate sexual orientation and gender identity in pediatric education settings remain lacking. The type of formal training that pediatric educators currently have for teaching of sexual orientation and gender identity is unclear and limited, which led to the development and implementation of this curriculum.

**Methods:**

A 2-hour workshop was developed to address gaps in knowledge, equip faculty and resident educators with skills to apply key concepts in teaching activities, and motivate them to examine challenges and opportunities in teaching sexual orientation and gender identity principles in their routine duties in pediatric settings across the undergraduate and graduate education spectrum. Learning strategies of the workshop included learner activation, a didactic, and clinical cases with role-play opportunities. Participants completed evaluations at the end of the workshop.

**Results:**

The workshop was implemented in three varied educational settings in 2019. All 65 participants enrolled in the workshop completed the evaluations. Evaluations ranged from 4.6 to 4.9 on a 5-point Likert scale (1 = *strongly disagree*, 5 = *strongly agree*). Participants reported workshop strengths and anticipated impact on their own teaching and clinical practice.

**Discussion:**

Stark health disparities for the LGBTQIA+ population and gaps in relevant curricula demand a training intervention for pediatric educators. We demonstrated the successful implementation of a training workshop, with evidence of feasibility and generalizability, that addressed knowledge gaps and teaching and clinical skills.

## Educational Objectives

By the end of this activity, learners will be able to:

1. Define key concepts of sexual orientation and gender identity as related to pediatric clinical care.
2. Apply these concepts to commonly encountered clinical scenarios related to sexual orientation and gender identity.
3. Identify challenges and opportunities for teaching sexual orientation and gender identity in routine clinical educational duties in the general, subspecialty, and acute care pediatric settings.

## Introduction

Significant health disparities continue to exist for the lesbian, gay, bisexual, transgender, queer, intersex, asexual, all other genders, sexes, and sexualities (LGBTQIA+) population in the United States, with sexual and gender minorities facing higher rates of HIV/AIDS, sexually transmitted infections (STIs), depression, suicide, and substance use disorders.^1,2^ Within the pediatric population, these disparities are disturbing, with close to a third of LGBTQIA+ minority youth reporting having attempted suicide, which is double the rate of their heterosexual, cisgender counterparts, 84% reporting verbal harassment, 30% being physically harassed, and increased rates of STIs among males having sex with males, which is in stark contrast to decreased rates of gonorrhea, chlamydia, and syphilis for all other adolescent groups.^3^

In recognition of these disparities, there has been a trend over the past few decades to increase cultural competency education around the care of LGBTQIA+ adults in undergraduate medical education. In 1991, a survey of all medical schools showed that a national average of 3 hours and 26 minutes was dedicated to LGBTQIA+ health across the 4-year curriculum.^4^ A more recent survey in 2011 showed an average of 5 hours dedicated to LGBTQIA+ health, but with almost a third of medical schools still reporting no time dedicated to the topic during clinical years.^5^ It is unclear what percentage of reported time in these surveys focused on the unique challenges of LGBTQIA+ youth. Furthermore, transgender health and gender-affirming care, specifically, are even more underrepresented in medical education.^2^ Some schools may have at most one course on this particular topic.^6,7^

Fewer cultural competency educational interventions on LGBTQIA+ health have focused on graduate medical education training programs, and even more specifically, on pediatric residents and faculty. A review of emergency medicine residency programs showed that only one in three programs included LGBTQIA+ health in the didactic curriculum.^8^ The lack of focus on residency training is particularly problematic, as residents often use this time to create skillsets that they continue to utilize for the remainder of their careers and also model learned skills to medical students during their clinical rotations. Similarly, though attending physicians may seek out continuing medical education trainings on LGBTQIA+ health, few opportunities exist to help train attending physicians on how to teach LGBTQIA+ health to residents and medical students. This presents as a missed opportunity to follow the medical education model of layered learning for trainees.

As stated by Miranda Hester, “providing gender-affirming care is necessary in pediatrics because the pediatrician is often the first medical professional in a child's journey to affirming gender identity and can set the tone for a lifetime of care.”^9^ Yet, there are insufficient formal curricula that target teaching this topic in pediatric settings to learners across the spectrum from medical students to faculty.^10^ In light of the health disparities that exist for LGBTQIA+ youth, we developed and implemented a curriculum for teaching sexual orientation and gender identity content to pediatric health care providers. While other interventions aimed to impart knowledge to learners,^7,10,11^ this workshop did more than target learners across the spectrum. It more uniquely employed a train-the-trainer approach, encouraging faculty and residents to not only become equipped with the knowledge and skills critical to their own patient care, but also to learn how to teach sexual orientation and gender identity content to others in clinical settings.

## Methods

### Curriculum Context and Target Audience

We recognized the need to develop this workshop after a literature review that included the Proceedings of the 2018 American Medical Women's Association Sex and Gender Health Education Summit which demonstrated the continued paucity of educational interventions.^8^ We based the content, methodology, and mode of delivery on literature findings, consensus opinions of expert pediatric medical educators, and principles of adult learning theory. Overall, we decided upon the content by a critique of what essential knowledge clinical educators need and what essential skills clinical educators need to develop to teach sex and gender content in direct pediatric patient care settings. The didactic presentation underwent rigorous review and was piloted at one author's institution. We used a lens of cultural humility for our cases, providing an opening for discussion and partnership with patients and their families rather than assuming a prescribed set of behaviors or values.

In recognition of the different needs of learners across the continuum, from generalists to specialists and medical trainees to nationally recognized leaders in pediatric LGBTQIA+ health, we created an author group with a diverse set of backgrounds including residents, general and subspecialist pediatricians, educators in health care communication, and adolescent medicine providers to help develop our training strategies. For our workshops, similarly, we chose facilitators with a wide range of specialties but with dedication and expertise in medical education and sex and gender minority populations. The facilitators participated in a detailed orientation regarding the overall content and instructional techniques of the workshop during two 1-hour conference call meetings. The facilitators used a facilitator guide (Appendix A) during the orientation meetings and while leading the workshop. All workshops contained the same content.

We did not require prior knowledge or skills for our participants of the workshop. We did inform participants that the workshop was a practical, interactive workshop. We sought to attract participants such as faculty and clinical educators with an interest in gaining knowledge and developing skills in the teaching of sexual orientation and gender identity content in clinical settings.

### Implementation

We delivered the highly interactive workshop during a 2-hour session, as detailed in the facilitator guide (Appendix A). We started the workshop with an introduction of the facilitators, who were then deliberately assigned to tables for facilitated work with the participants. We then engaged the learners with a learner activation component, which included the showing of a 7-part patient vignette video (Appendix B) and a needs assessment of the participants. The video revealed the gender-affirming medical care experiences of a patient and his mother since his early childhood. During the needs assessment, participants were asked to consider: (1) What is the current state of affairs at your institution?, (2) What are some challenges that you face in teaching of this content domain?

A didactic PowerPoint presentation (Appendix C) followed, presenting an overview of sexual orientation and gender identity content as it pertained to pediatric patient care and clinical educators involved in direct patient care teaching. The presentation contained all the knowledge content needed to meet the learning objectives. We continued learner engagement with an introduction of selected education resources (Appendix D) for learners to use both in the workshop and at their institutions. These materials reflected the didactic session's content.

Learners then engaged in a series of small-group case-based discussions and relevant role-plays (45 minutes), which highlighted the key content and directly linked to the workshop's learning objectives. Four cases with optional role-plays (Appendix E) were developed with both learning process and content expert review. Cases were deliberately developed to represent adolescent medicine, specialty care, and general pediatric care teaching scenarios, which highlight key tenets of sexual orientation and gender identity content. Facilitator's used key talking and scripted points to highlight the content and skills that linked to the learning objectives. The role-plays were intended to allow the learner to practice the skills and delivery of knowledge that was stressed in the proceeding related case discussion. Based on time constraints, not all workshop groups reviewed all four cases. A 15-minute large-group debrief session followed the case discussions to further participants’ awareness of their role as clinical teachers and their application of content principles in clinical teaching settings.

Finally, participants separated into think-pair-share groups to discuss and reflected upon missed teaching opportunities in their own teaching activities for 10 minutes. We ended the workshop with a 15-minute summary of the lessons learned and a discussion of access to pertinent content material and other resources to be used in participants’ clinical teaching duties.

### Preparation of the Workshop Space

The activity required:

• Round tables for six to 10 participants.
• Audiovisual support including an LCD projector.
• Two flip charts at the front of the room.
• Badge material for learners and workshop facilitators to identify their correct pronouns.

We ensured that there were enough facilitators to establish a good ratio of facilitator to learners (i.e., 1:3–5) at each table to explore the content of each case. To stay on schedule, we designated one workshop leader to serve as the timekeeper.

### Materials for Workshop Facilitators

We provide materials formatted for a 2-hour workshop. We did not require that all workshop facilitators hold content expertise. The guides and references included in this publication were designed to provide typical medical educators with the background needed to meaningfully serve as facilitators. We encouraged facilitators to engage in self-study of the materials, including the references, and follow up with group discussions to ensure that all facilitators had similar and sufficient understanding prior to implementation of the workshop.

### Evaluation Form

We distributed an anonymous paper evaluation form (Appendix F) to all participants at the conclusion of each workshop. The evaluation form used a 5-point Likert scale (1 = *strongly disagree*, 5 = *strongly agree*) to rate the overall effectiveness of the workshop and to address the three specific learner objectives. The evaluation form was uniquely developed to obtain learners’ self-reports regarding their perceived knowledge and clinical skills gains. We also created open-narrative response questions to allow for detailed answers about how learners met the educational objectives and to examine another dimension of the learners’ experiential learning. The evaluation form overall was intended to capture self-reported learner outcomes achieved during the actual workshop as well as learners’ plans for further work outside of the workshop. The narrative responses also provided feedback for the facilitators for iterative improvement of subsequent workshops and the final forms of the facilitator guide and case discussions presented in this publication (Appendices A and E).

## Results

The workshop was successfully implemented at the Pediatric Academic Societies (PAS) meeting in Baltimore, Maryland in April 2019, and at residency programs at the University of California (UC) Davis Medical Center in Davis, California in June 2019, and Baylor College of Medicine—The Children's Hospital of San Antonio (BCM-CHofSA) in San Antonio, Texas in August 2019.

The learners at PAS consisted of 10 pediatric faculty and three pediatric residents, whose experience in medical education varied across the learner continuum and across primary and specialty fields. The learners chose to attend the workshop from a list of other concurrent workshops at the meeting. The learners at UC Davis and BCM-CHofSA consisted of 19 pediatric residents and faculty and 33 pediatric residents, respectively. The learners participated in the workshop as part of their core residency curriculum.

All 65 learners who attended the workshops at PAS, UC Davis, and BCM-CHofSA completed the postworkshop evaluation forms. Table 1 shows the prompts, respondent numbers, and mean Likert-scale responses (1 = *strongly disagree*, 5 = *strongly agree*). Learners rated the workshops favorably for all prompts on the evaluation form, with the highest rated prompts being, “I learned new knowledge and skills from this activity,” and, “I will apply the knowledge and skills from this activity,” which both received a mean rating of 4.8 when participant responses from all three workshops were combined.

**Table 1.**
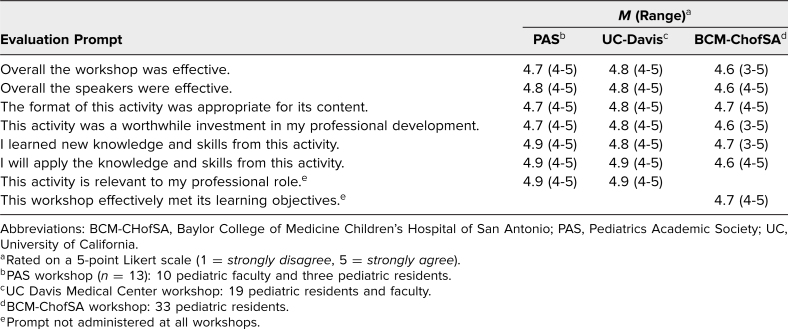
Participant (*N* = 65) Responses to Postworkshop Evaluations from Three Educational Meetings

Table 2 summarizes representative narrative responses. No thematic differences were noted between the three implementation sites or between the two learner groups of faculty and residents. Both learner groups frequently identified that they intended to make improvements in their communication skills, including appropriate inquiry with patients and families, proper use of pronouns, and working with electronic medical records. Both learner groups also identified clinical practice improvements they planned to make including how they conduct their home, education, peer group activities, drugs, sexuality, and suicide/depression (HEADSS) assessments and model history-taking and physical exam with learners in direct patient-care settings. Resident learners also emphasized how they will incorporate referrals to resources to support transgender patients and recommend provision of a list of local resources for this patient population.

**Table 2.**
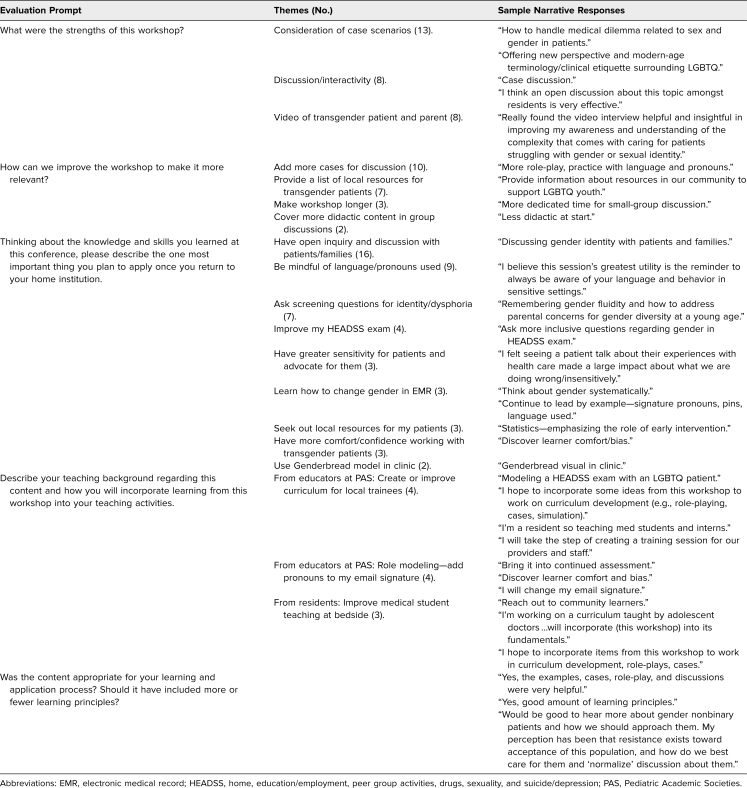
Participant (*N* = 65) Narrative Responses on Postworkshop Evaluations at Three Educational Meetings

## Discussion

While there has been some recent emphasis on LGBTQIA+ health with a focus on sexual orientation and gender identity related to pediatric care, educators still have little training in teaching this topic in clinical settings.^4–6,10^ A deliberate effort to integrate LGBTQIA+ health as a global thread in education opportunities and continuing medical education is crucial to impact patient outcomes. However, there remains a gap in the medical education literature with regards to training educators to teach this important educational content. The training gap is stark in the literature from the perspective of both patients and learners.^1–6^ We offer a workshop that addresses gaps in knowledge and clinical skills in this critical health care topic while using the train-the-trainer approach.

Our workshop offers several distinct features. First, our work focused on the pediatric clinical setting and pediatric learners in contrast to most of the previous work in the literature on LGBTQIA+ health, which has focused on adult patient care.^2,11^ Secondly, most interventions such as Gallego and Knudsen aim to only disseminate direct knowledge.^7,11,12^ Our workshop was designed to provide faculty and resident the knowledge and skills needed to not only deliver appropriate care themselves, but also to teach sexual orientation and gender identity principles and LGTBQIA+ health to medical students and residents in clinical settings. Indeed, developing residents as teachers has become an emphasis.^13,14^ We are not aware of other learning interventions that target residents as teachers of this important health care topic. Finally, our workshop is one of few educational interventions on LGBTQIA+ health whose content and strategies are based on the input of both content experts and a wide variety of education experts in both learner and faculty development.

Our workshop demonstrated feasibility, adaptability, and generalizability with successful implementation for both resident and faculty learners at three different learning sites. The clinical content was broad yet practical, and overall was highly valued by most learners. It can be challenging to target key clinical learning points regarding a patient population with vast and potentially overwhelming needs and disparities. Nevertheless, our workshop succeeded in highlighting key patient care points. The content had universal clinical relevance with a high number of participants agreeing that the workshop will help them have open inquiry and discussion with patients and their families in teaching settings—a crucial tenet in patient communication. Of note, clinical educators particularly expressed this tenet in their narrative evaluations of the workshop. Indeed, the workshop promoted a train-the-trainer model and helped facilitate faculty themselves to address their own gaps in knowledge and clinical skills as they learned how to teach the pertinent knowledge and skills to their own learners. This type of model of stealth curriculum is often advantageous to critical topics in which faculty may assume they already have facility.

Our workshop can be adapted to a variety of learning environments. The needs of learners and the needs of the patients they serve—and will serve—should make the need for faculty development obvious. Nevertheless, institutions need to be willing to consider this curriculum intervention, whose content is still relatively new in pediatric medical education. Having a workshop such as ours, which can be adapted to varying learner needs and other contexts, will help curb some of the challenges in meeting this educational need. That the workshop held expert reviewed and tested content with rigorous adherence to peer review standards in medicine should help decrease barriers to teaching and developing faculty who feel less comfortable with this important content.

### Limitations

Terminology in this content area is rapidly evolving. One limitation regarding content was that our teaching materials originally used terminology that has now been replaced with more inclusive phrases. At the time of publication, we have updated all resources presented to include only affirming language that is the standard of care. Aiming to be culturally humble providers, we recognize that terminology will continue to evolve, and caution those using our resources to ensure the language utilized is up to date with other materials they may be referencing prior to implementing our curriculum verbatim. The link and resources offered in Appendix D are often updated and are recommended for educators to review for any updates to proper language and usage.

Secondly, the cases were not intended to be inclusive of all LGBTQIA+ topics, but instead offered a starting point for conversation and teaching. In addition, we did not feature the full range of sexual and gender minority communities in our content. Finally, although the workshop was shown to impact knowledge, skills, and attitudes of participants to date by their report, we have yet to study our curriculum to evaluate actual changes in knowledge or practice. However, given the importance of this area and its truly innovative approach to an evolving topic, we share this curriculum as a framework for future work and outcomes-based research. Our self-reported outcomes likely attested not only to learners meeting the workshop's objectives, but also endorsed the overall need for this type of faculty development in a clinical care topic that is rapidly being recognized as critically important to patient care.^9^

### Future Directions

While the evaluation outcomes reinforced the feasibility and adaptability of the workshop, the next step in the evaluation process is to assess its impact on learner behaviors at a higher level in Kirkpatrick's hierarchy.^15^ Participants have already indicated behaviors they intended to change following the workshop. In the future, we plan to study higher-level outcomes such as demonstrated gains in knowledge and skills. We encourage future facilitators to seek higher outcomes measures as they implement this workshop at their institutions and in other venues. This will allow us to lead the next generation of medical trainees to have a greater impact on patient care for this important and vulnerable patient population.

### Conclusion

In summary, disparities in LGTBQIA+ health described in the literature are dramatic, particularly among youth. It is also clear that formal education interventions regarding critical knowledge and clinical skills for medical students and residents are lacking. Strikingly, little is known regarding the education that clinical educators and faculty themselves have received on this topic. Education leaders and clinical educators should aim to teach and model the appropriate knowledge and clinical skills with their learners. This workshop addressed both the needs of clinical educators and learners by providing faculty and residents the knowledge and skills required to deliver appropriate care themselves and to teach sexual orientation, gender identity, and LGTBQIA+ health to others in clinical settings.

## Appendices

Facilitator Guide.docxPatient Vignettes.pptxDidactic Presentation.pptxSelected Educational Resources.docxCase Discussion with Role-Play Opportunities.docxEvaluation Form.docx
All appendices are peer reviewed as integral parts of the Original Publication.
