# Supplementary material for: Teaching Sexual Orientation and Gender Identity in Pediatric Clinical Settings: A Training Workshop for Faculty and Residents
Source: MedEdPORTAL. 2021 Apr 5;17:11137. doi: 10.15766/mep_2374-8265.11137 (PMC8034234; doi:10.15766/mep_2374-8265.11137)
Supplement: Supplementary file 1 — Facilitator Guide.docxPatient Vignettes.pptxDidactic Presentation.pptxSelected Educational Resources.docxCase Discussion with Role-Play Opportunities.docxEvaluation Form.docx [file mep_2374-8265.11137-s001.zip › A. Facilitator Guide.docx]

# Appendix A: Facilitator Guide

### Learning objectives for the workshop

By the end of this activity, learners will be able to:

1. Define key concepts of sexual orientation and gender identity as related to pediatric clinical care.
2. Apply these concepts to commonly encountered clinical scenarios related to sexual orientation and gender identity.
3. Identify challenges and opportunities for teaching sexual orientation and gender identity in routine clinical educational duties in the general, subspecialty, and acute care pediatric settings.

While sexual orientation and gender identity issues in pediatrics have risen to some prominence, a more deliberate effort to integrate it as a global thread in more pediatric education opportunities is needed.

This training workshop aims to address gaps in knowledge, to equip educators with skills to apply key concepts to their teaching efforts and to have educators examine their challenges and opportunities to teach sexual orientation and gender identity issues in pediatrics in their routine clinical educational activities in pediatrics. Learning strategies include: learner activation, a didactic, and intentionally developed cases and roleplays to focus on teaching opportunities in both general and specialist pediatrics settings. Participants will complete evaluations at the end of the training sessions.

| Time | Section | Content | Technique | Obj # | Facilitator |
| --- | --- | --- | --- | --- | --- |
| 5 min | Introduction | - Leader Introductions - Goals & Objectives | Large Group |  |  |
| 10  min | Learner Activation | These exercises will explore participants’ perceptions and experiences of this topic, their “current state of affairs” and barriers to their own teaching, and reinforce the global nature of this content in clinical teaching.  It will be especially noted that the appropriate nomenclature and content is evolving – maintain a lens of cultural humility | - Online Poll (optional)   Consider: 1. What is the “current state of affairs” at your institution? 2. What are some challenges that you face in teaching of this content domain?   - Video - Large Group *Call-Out “*Current State of Affairs” | 2,3 |  |
| 20  min | Didactic | ***Overview*** of Key Principles  Key, sentinel content to update our participants – What is known at this time for “Best Practice”  The didactic will stress that its teaching points are not inclusive of all topics and will direct the learner to best-practice and peer-reviewed resources. | Didactic  ***Time allocation can be adapted to the venue and to the learner group. | 1-3 |  |
| 45  min | **Teaching and Patient Care**  (Pick 3 cases)  1. Case-based discussions  2. Role plays | - Practice Opportunities - Application of content principles in various clinical settings - *“How could we make this a good teaching moment for our learners?”*   *** These cases do not represent the full breadth and depth of LGBTQIA+ issues such as sexually transmitted infections or contraceptive discussions. It is recommended that workshop leaders address this lack of full inclusivity at the start of the session. | - Case-based discussions - Cases to “Train the Trainor” - Role Playing-optional - Consider Triad- role plays | 1-3 |  |
| 15  min | **Large Group Call out on Cases/Role plays- share from each table** | - Open further participants’ awareness of their role as clinical teachers. - Application of content principles in various clinical settings |  | 2,3 |  |
| 10  min | **Summary Missed Opportunities** | Exercise to deliberate on missed opportunities in their own teaching activities | Think-Pair-Share | 2,3 |  |
| 15 min | **Summary of take-home next-step pearls**  **Access to pertinent materials**  **List of resources**  **Wrap Up/Evals** | Activity to summarize lessons learned. Participants will receive a summary of take-home, next-step pearls, access to pertinent content material and other resources to be used in their clinical teaching duties. | Large Group Call Out | 1-3 |  |
