# Supplementary material for: Teaching Sexual Orientation and Gender Identity in Pediatric Clinical Settings: A Training Workshop for Faculty and Residents
Source: MedEdPORTAL. 2021 Apr 5;17:11137. doi: 10.15766/mep_2374-8265.11137 (PMC8034234; doi:10.15766/mep_2374-8265.11137)
Supplement: Supplementary file 1 — Facilitator Guide.docxPatient Vignettes.pptxDidactic Presentation.pptxSelected Educational Resources.docxCase Discussion with Role-Play Opportunities.docxEvaluation Form.docx [file mep_2374-8265.11137-s001.zip › C. Didactic Presentation.pptx]

## Slide 1
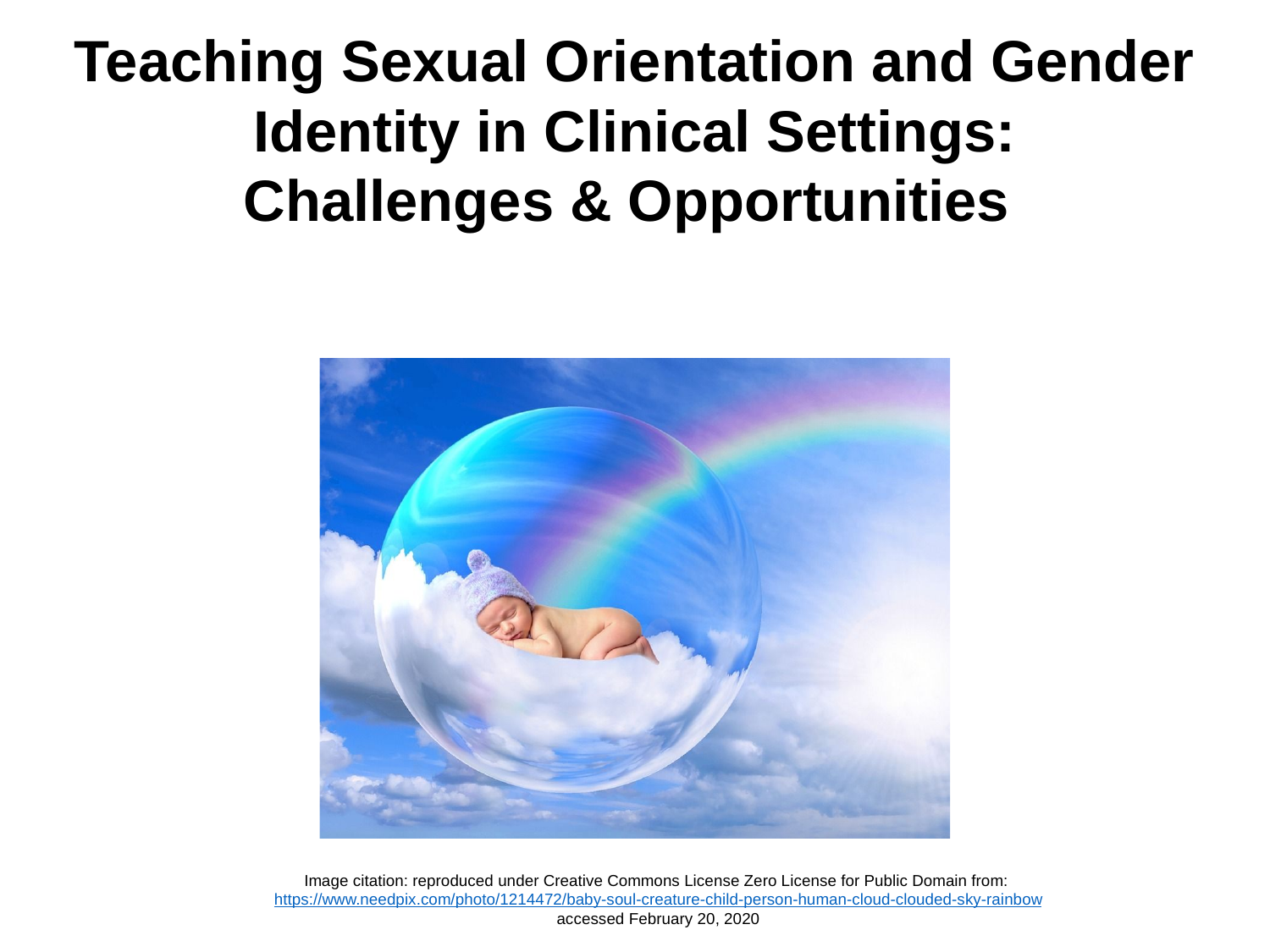

# Teaching Sexual Orientation and Gender Identity in Clinical Settings: Challenges & Opportunities
Image citation: reproduced under Creative Commons License Zero License for Public Domain from: https://www.needpix.com/photo/1214472/baby-soul-creature-child-person-human-cloud-clouded-sky-rainbow
accessed February 20, 2020

## Slide 2
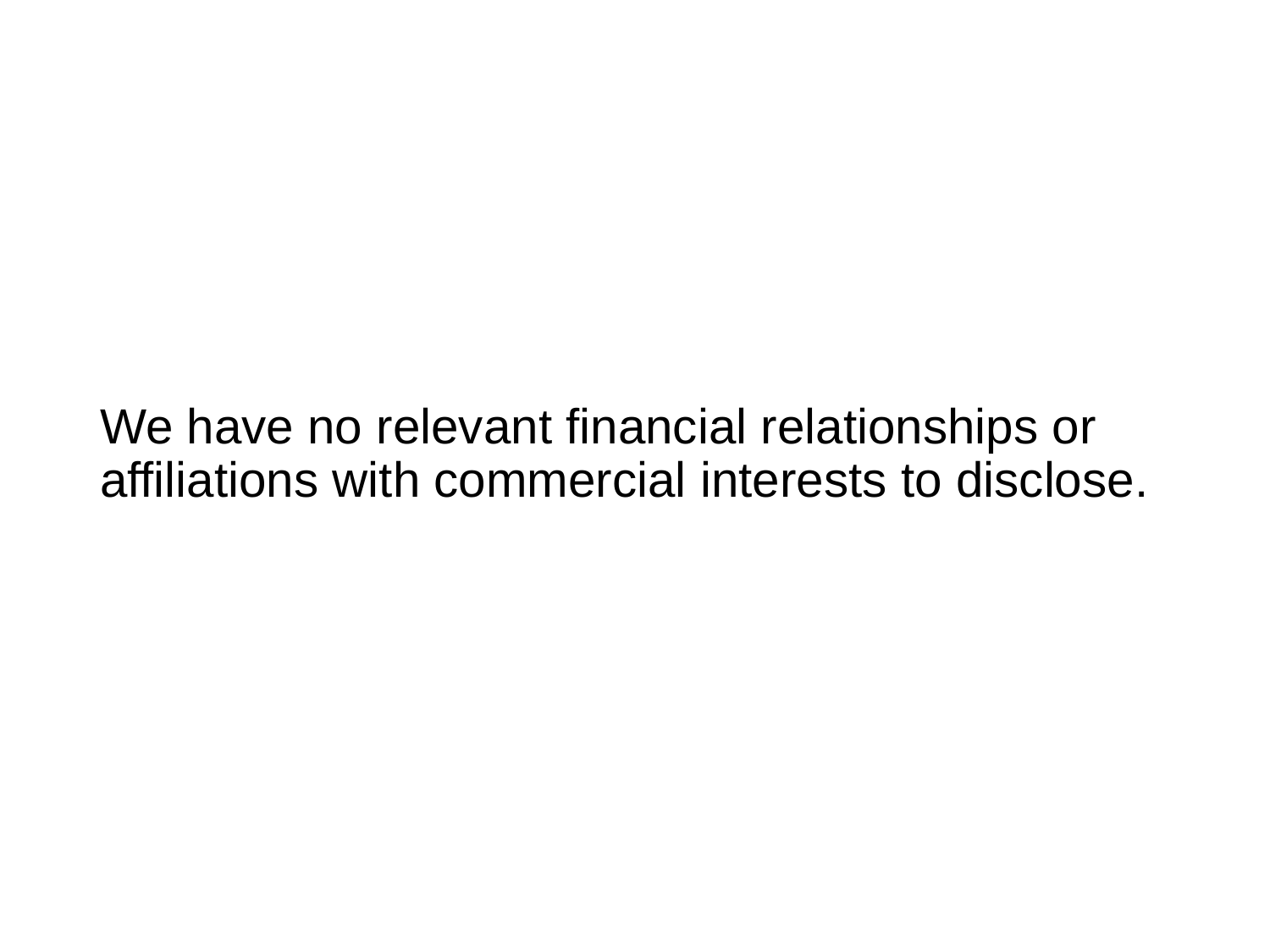

#
We have no relevant financial relationships or affiliations with commercial interests to disclose.

## Slide 3
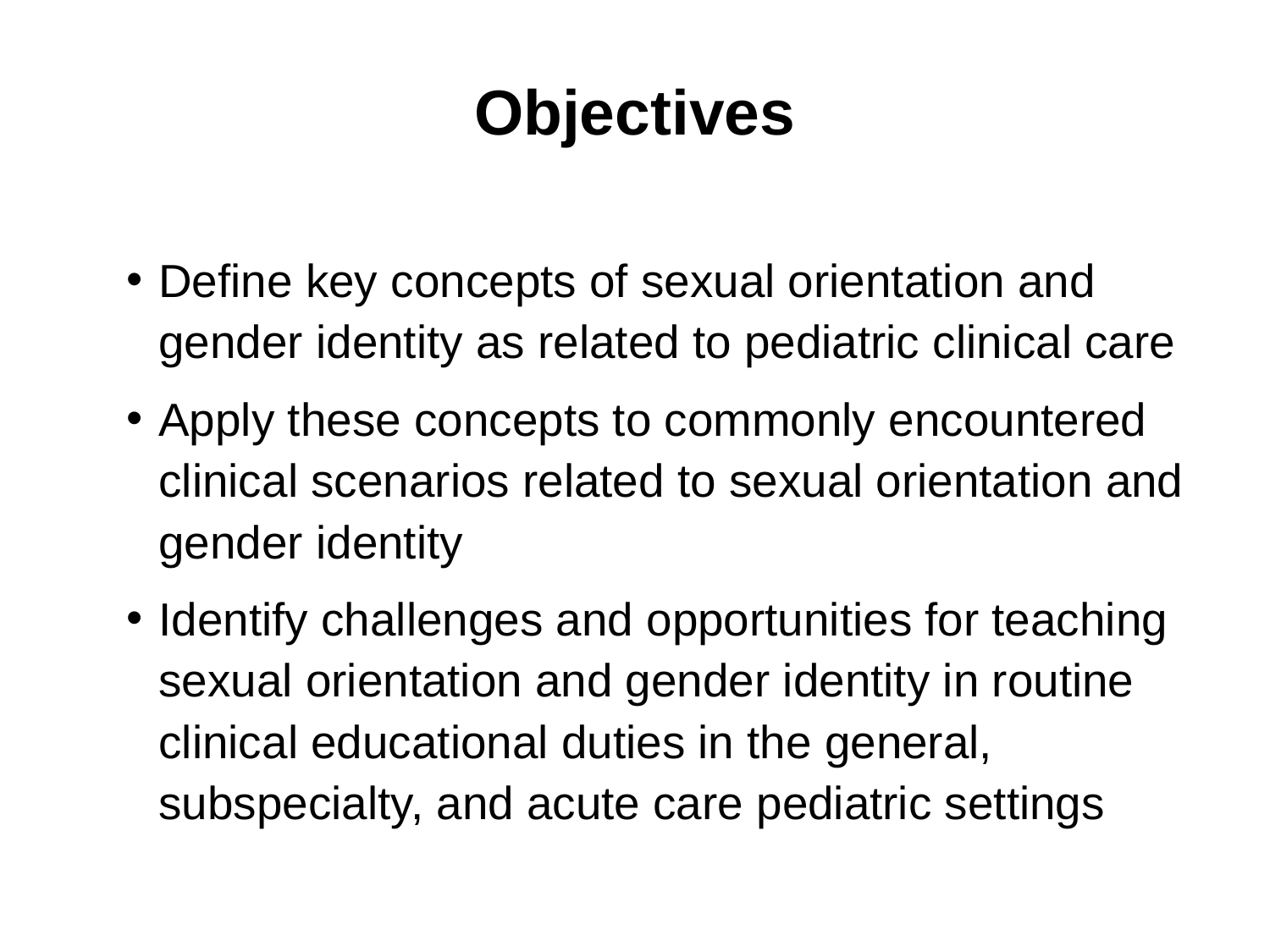

# Objectives
Define key concepts of sexual orientation and gender identity as related to pediatric clinical care
Apply these concepts to commonly encountered clinical scenarios related to sexual orientation and gender identity
Identify challenges and opportunities for teaching sexual orientation and gender identity in routine clinical educational duties in the general, subspecialty, and acute care pediatric settings

## Slide 4
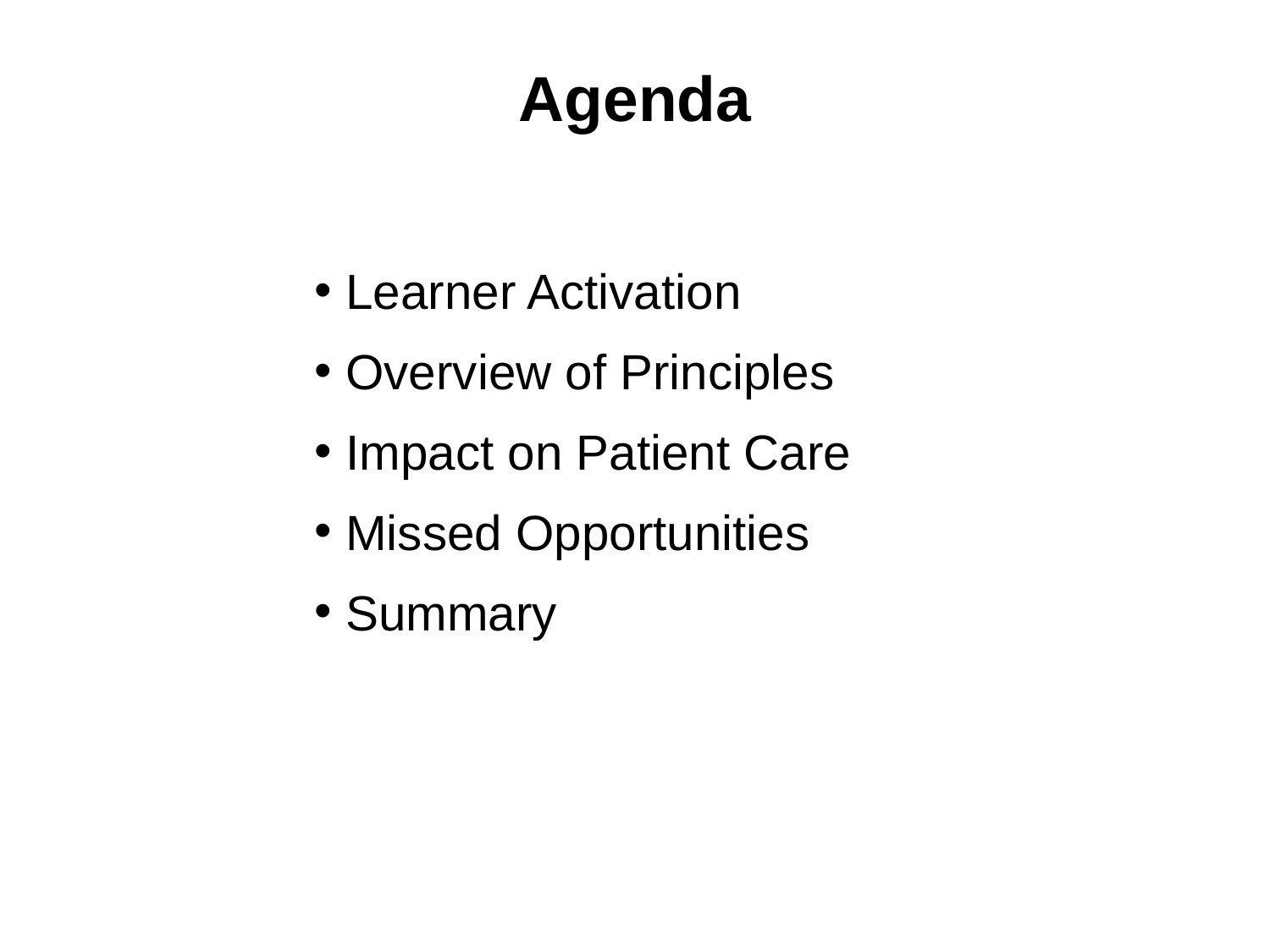

# Agenda
Learner Activation
Overview of Principles
Impact on Patient Care
Missed Opportunities
Summary

## Slide 5
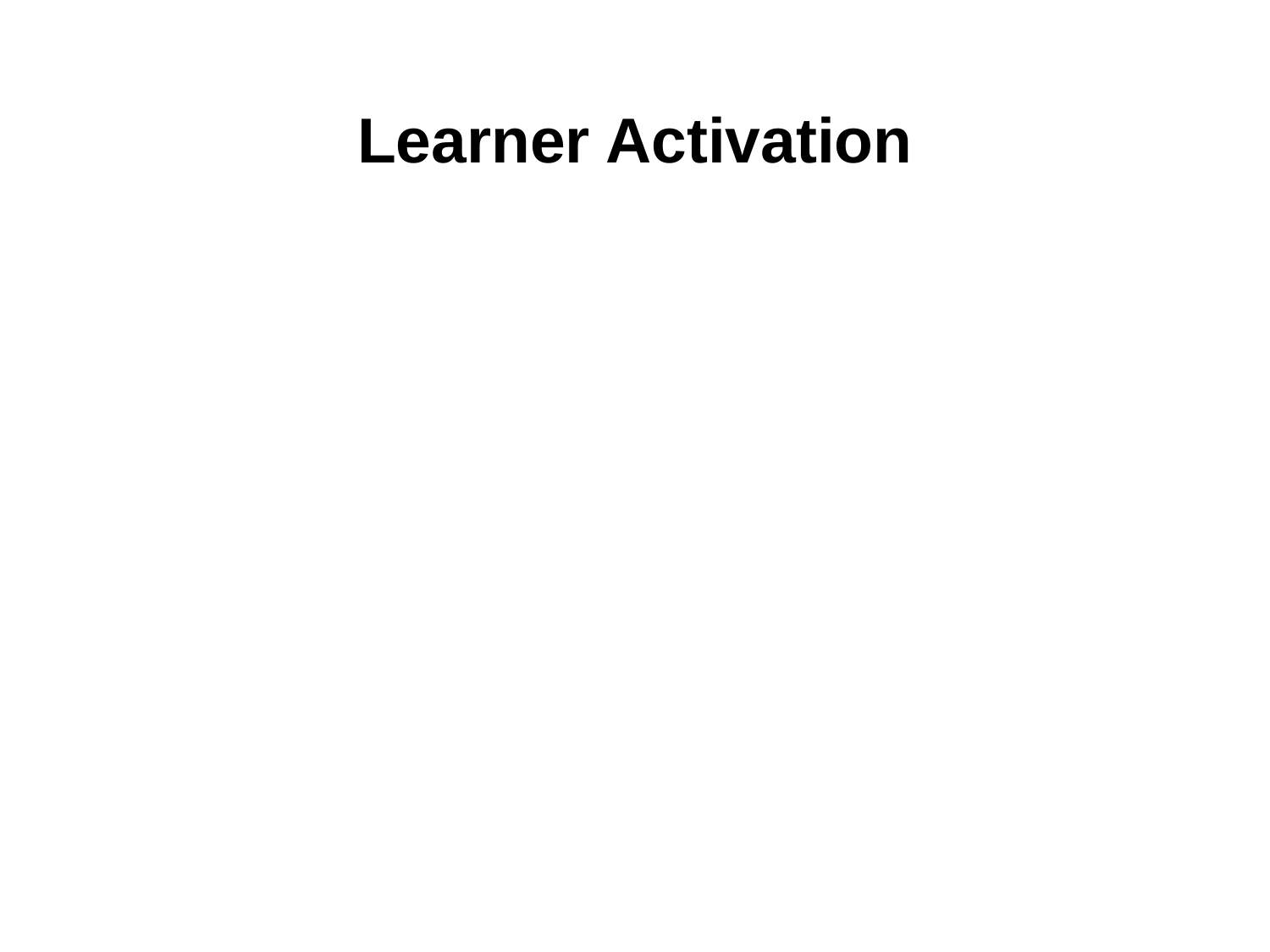

# Learner Activation

## Slide 6
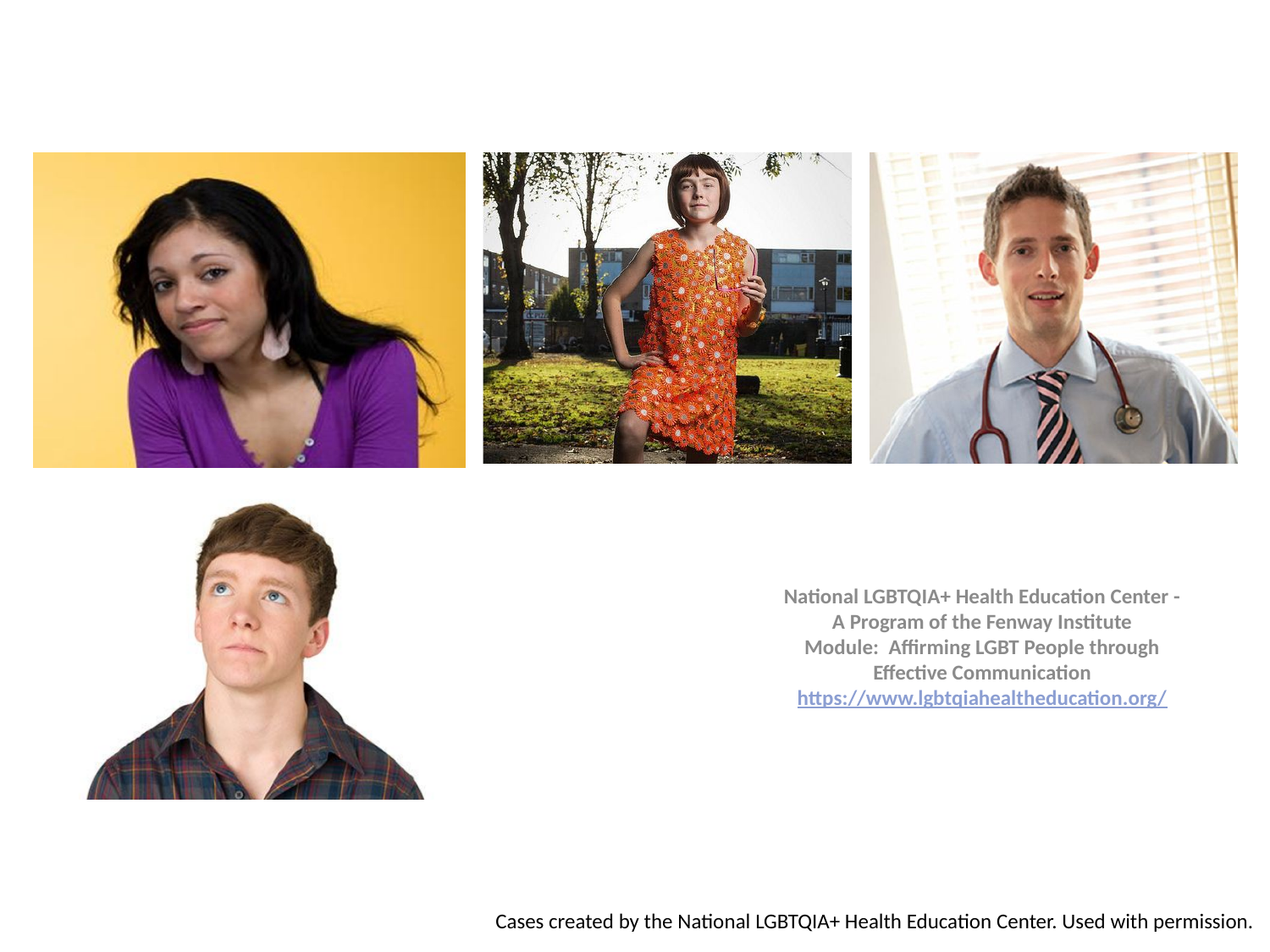

# Real life scenarios
National LGBTQIA+ Health Education Center - A Program of the Fenway Institute
Module: Affirming LGBT People through Effective Communication
https://www.lgbtqiahealtheducation.org/
Cases created by the National LGBTQIA+ Health Education Center. Used with permission.

## Slide 7
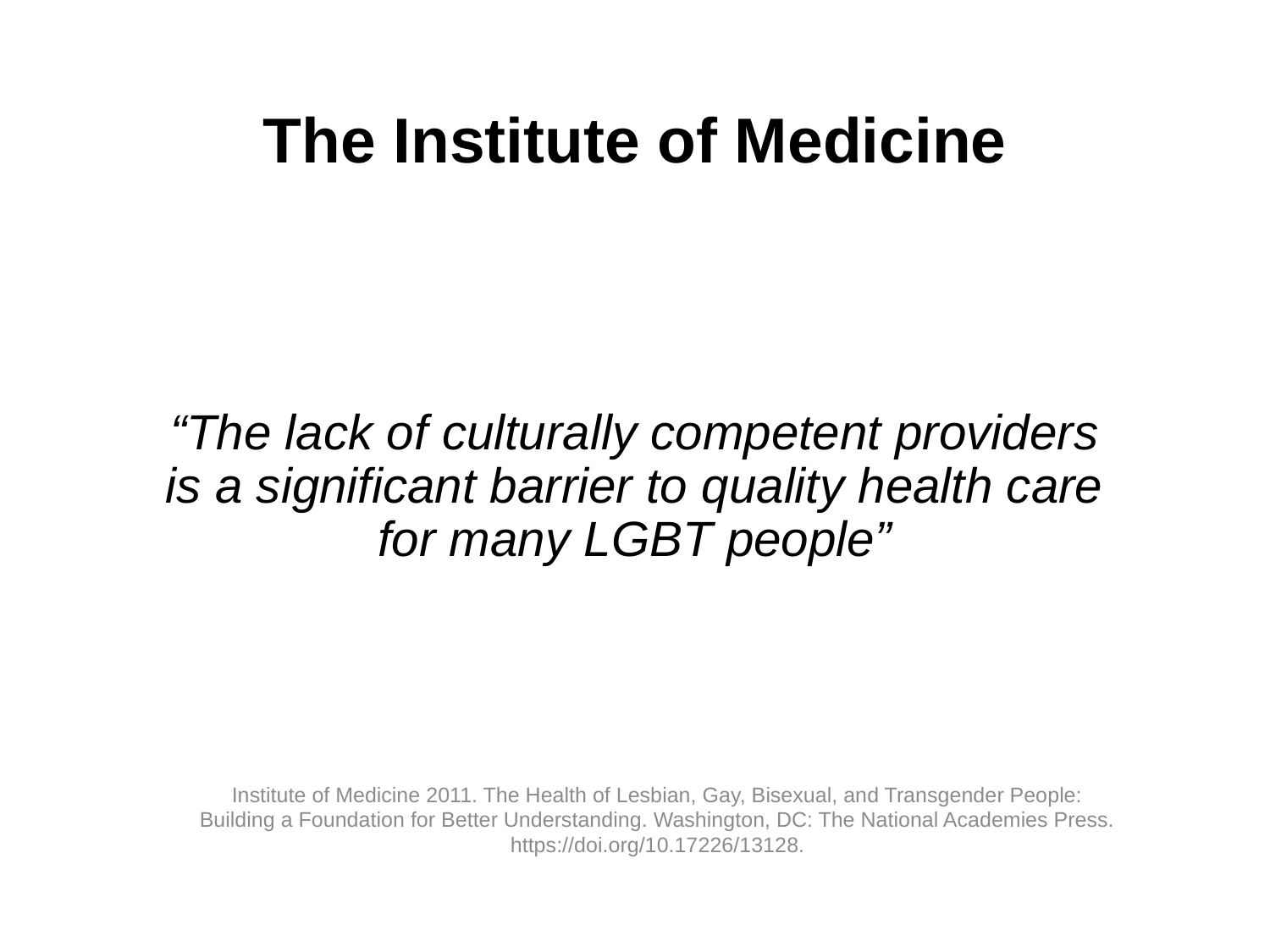

# The Institute of Medicine
“The lack of culturally competent providers
is a significant barrier to quality health care
for many LGBT people”
Institute of Medicine 2011. The Health of Lesbian, Gay, Bisexual, and Transgender People:
Building a Foundation for Better Understanding. Washington, DC: The National Academies Press. https://doi.org/10.17226/13128.

## Slide 8
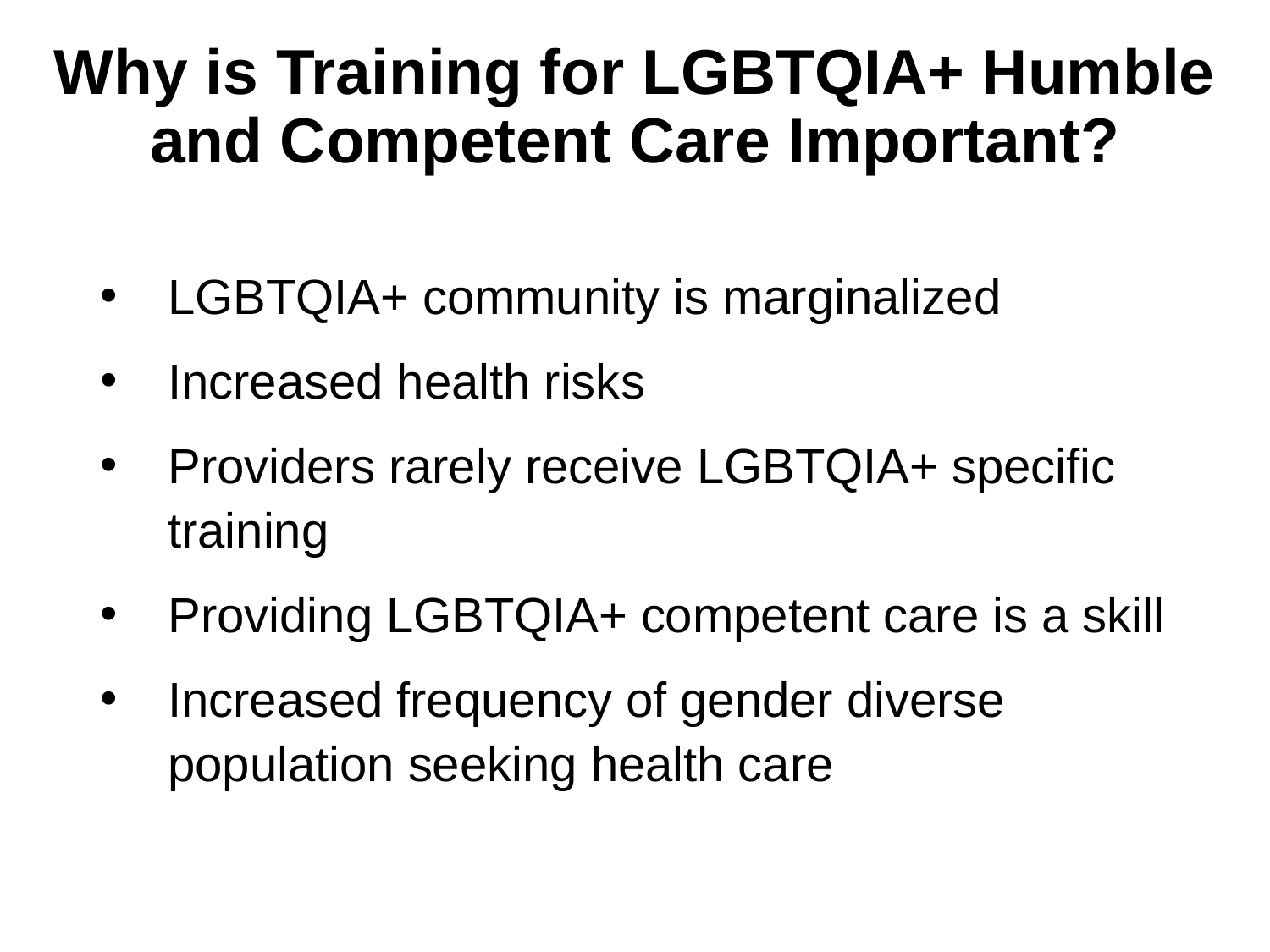

# Why is Training for LGBTQIA+ Humble and Competent Care Important?
LGBTQIA+ community is marginalized
Increased health risks
Providers rarely receive LGBTQIA+ specific training
Providing LGBTQIA+ competent care is a skill
Increased frequency of gender diverse population seeking health care

## Slide 9
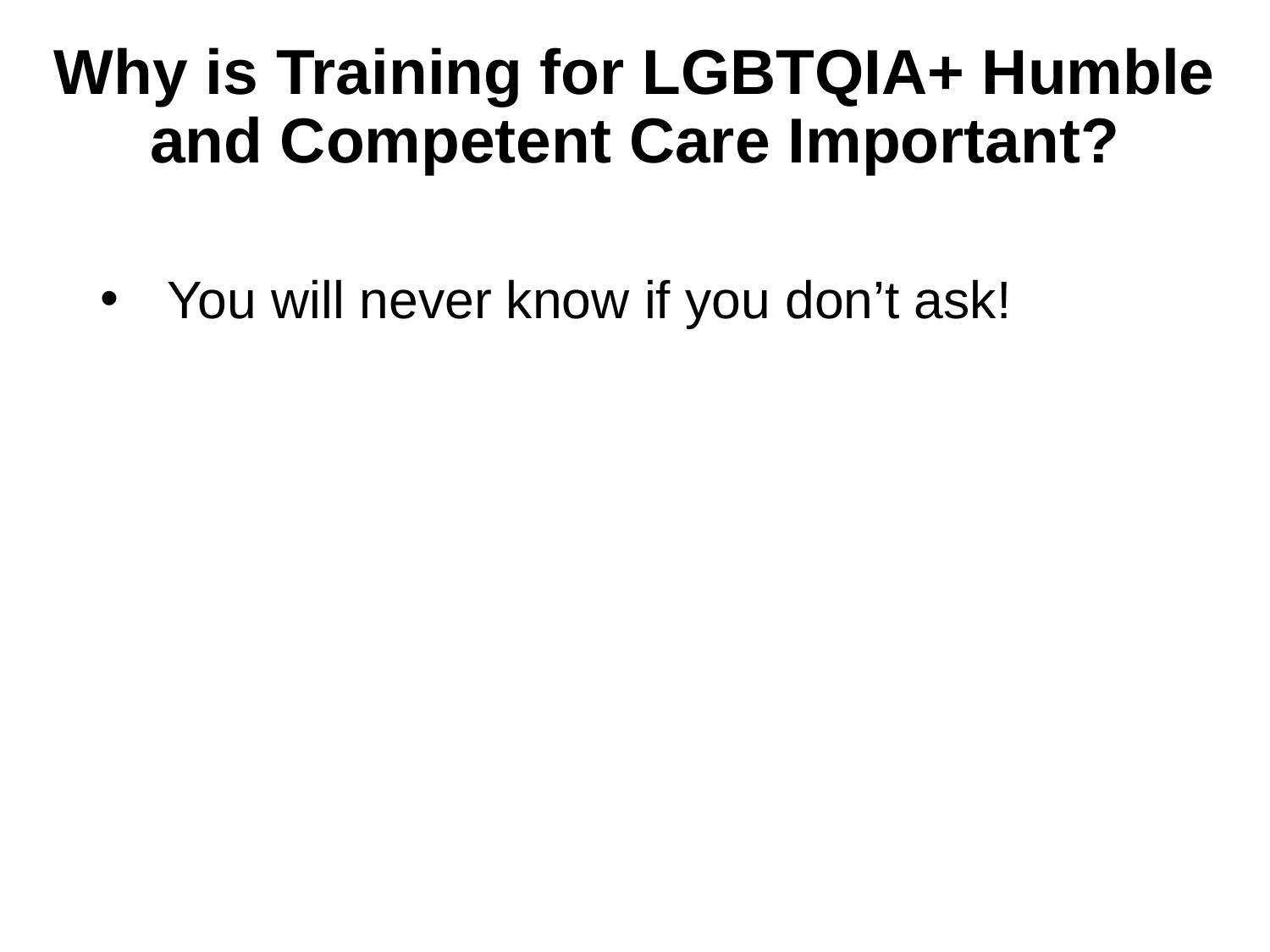

# Why is Training for LGBTQIA+ Humble and Competent Care Important?
You will never know if you don’t ask!

## Slide 10
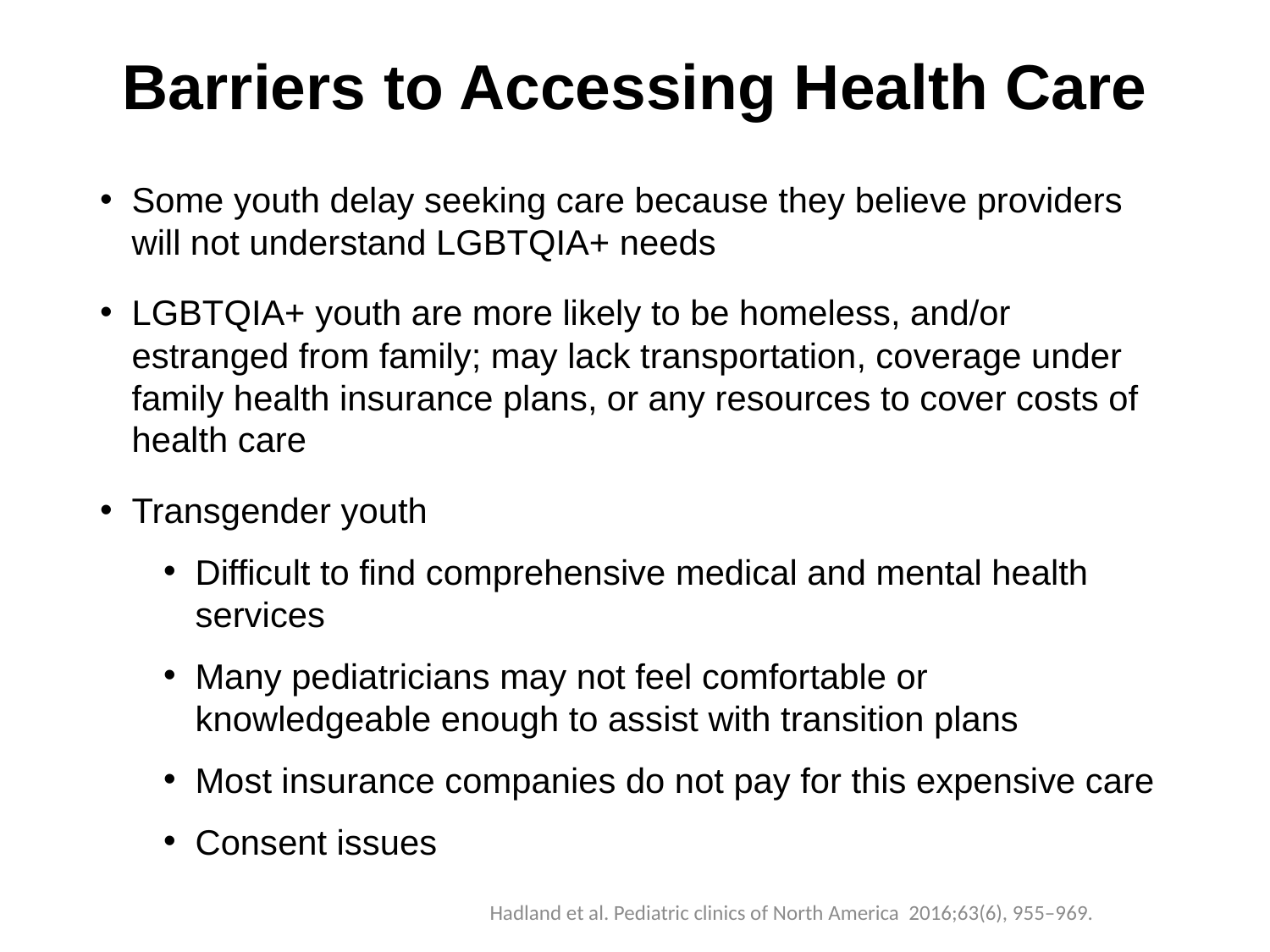

# Barriers to Accessing Health Care
Some youth delay seeking care because they believe providers will not understand LGBTQIA+ needs
LGBTQIA+ youth are more likely to be homeless, and/or estranged from family; may lack transportation, coverage under family health insurance plans, or any resources to cover costs of health care
Transgender youth
Difficult to find comprehensive medical and mental health services
Many pediatricians may not feel comfortable or knowledgeable enough to assist with transition plans
Most insurance companies do not pay for this expensive care
Consent issues
Hadland et al. Pediatric clinics of North America 2016;63(6), 955–969.

## Slide 11
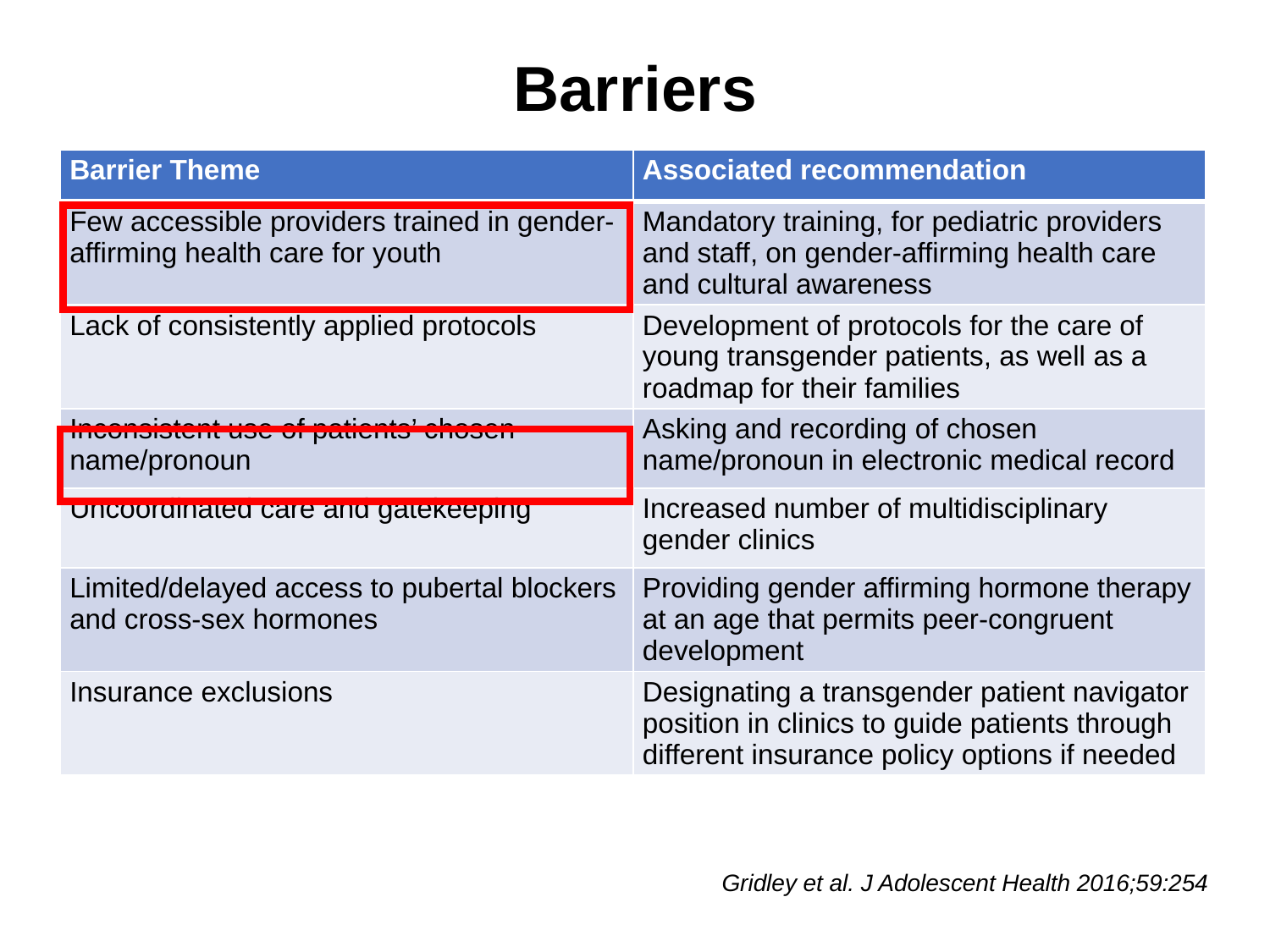

# Barriers
| Barrier Theme | Associated recommendation |
| --- | --- |
| Few accessible providers trained in gender-affirming health care for youth | Mandatory training, for pediatric providers and staff, on gender-affirming health care and cultural awareness |
| Lack of consistently applied protocols | Development of protocols for the care of young transgender patients, as well as a roadmap for their families |
| Inconsistent use of patients’ chosen name/pronoun | Asking and recording of chosen name/pronoun in electronic medical record |
| Uncoordinated care and gatekeeping | Increased number of multidisciplinary gender clinics |
| Limited/delayed access to pubertal blockers and cross-sex hormones | Providing gender affirming hormone therapy at an age that permits peer-congruent development |
| Insurance exclusions | Designating a transgender patient navigator position in clinics to guide patients through different insurance policy options if needed |
Gridley et al. J Adolescent Health 2016;59:254

## Slide 12
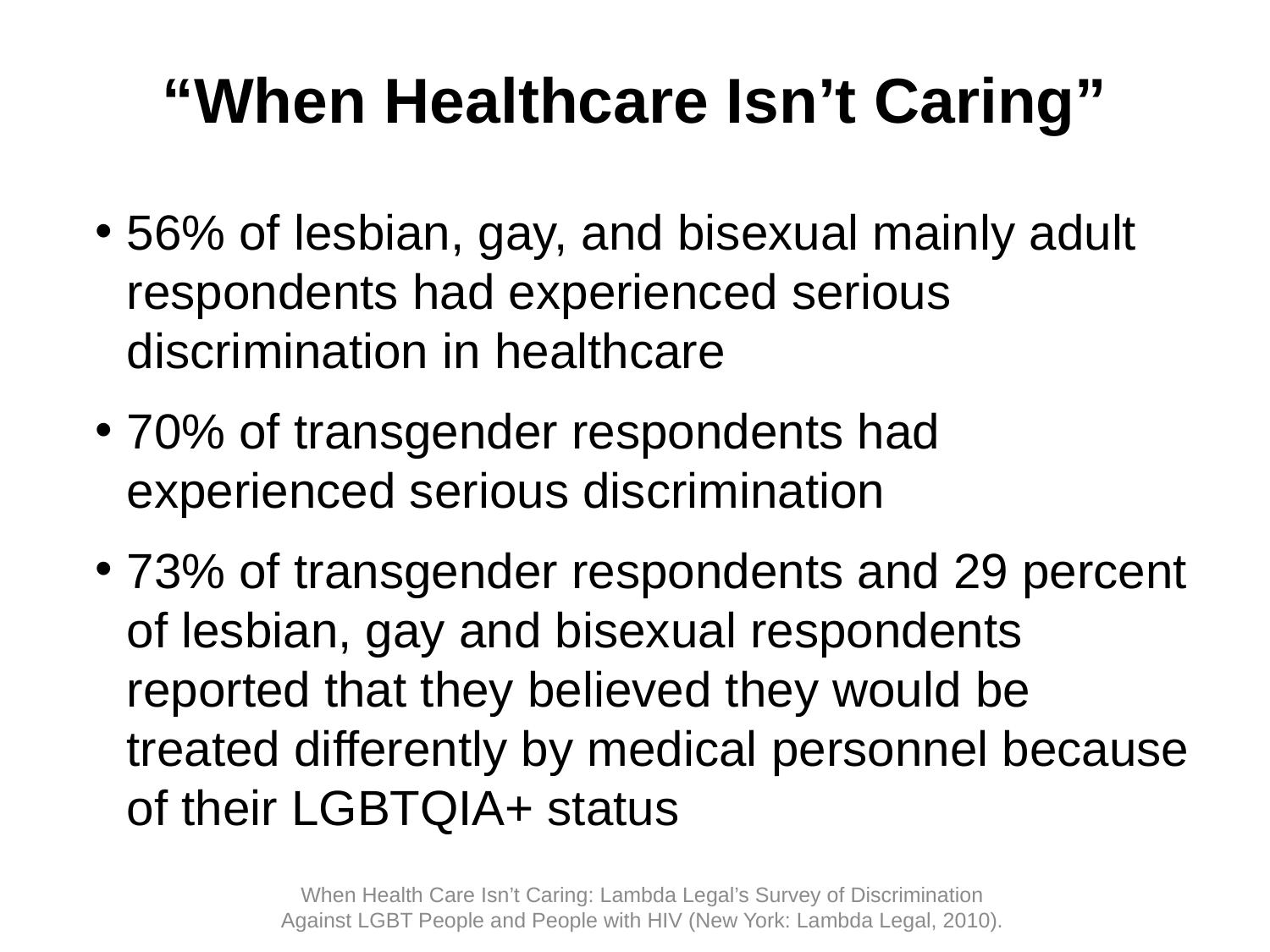

# “When Healthcare Isn’t Caring”
56% of lesbian, gay, and bisexual mainly adult respondents had experienced serious discrimination in healthcare
70% of transgender respondents had experienced serious discrimination
73% of transgender respondents and 29 percent of lesbian, gay and bisexual respondents reported that they believed they would be treated differently by medical personnel because of their LGBTQIA+ status
When Health Care Isn’t Caring: Lambda Legal’s Survey of Discrimination
Against LGBT People and People with HIV (New York: Lambda Legal, 2010).

## Slide 13
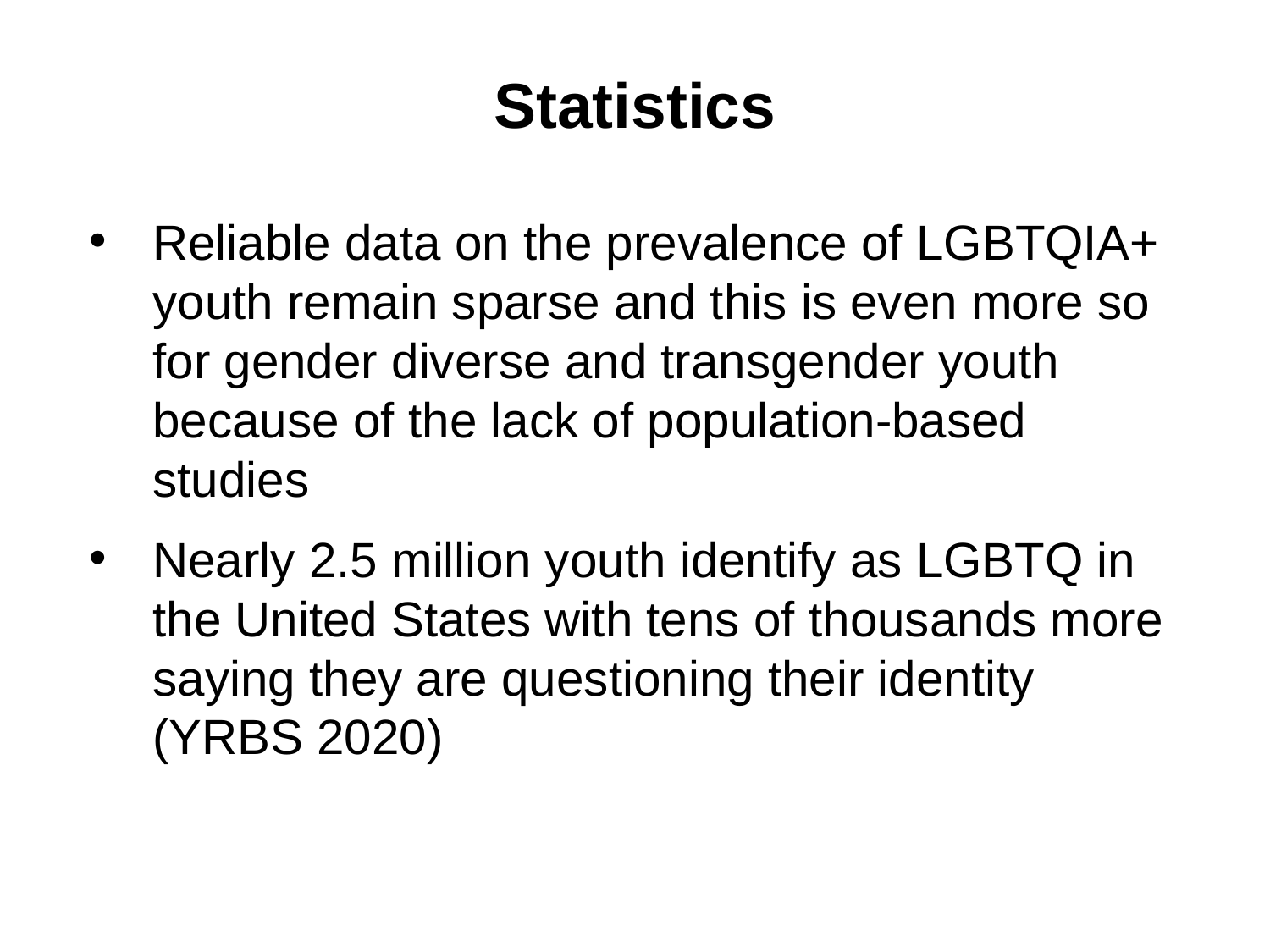

# Statistics
Reliable data on the prevalence of LGBTQIA+ youth remain sparse and this is even more so for gender diverse and transgender youth because of the lack of population-based studies
Nearly 2.5 million youth identify as LGBTQ in the United States with tens of thousands more saying they are questioning their identity (YRBS 2020)

## Slide 14
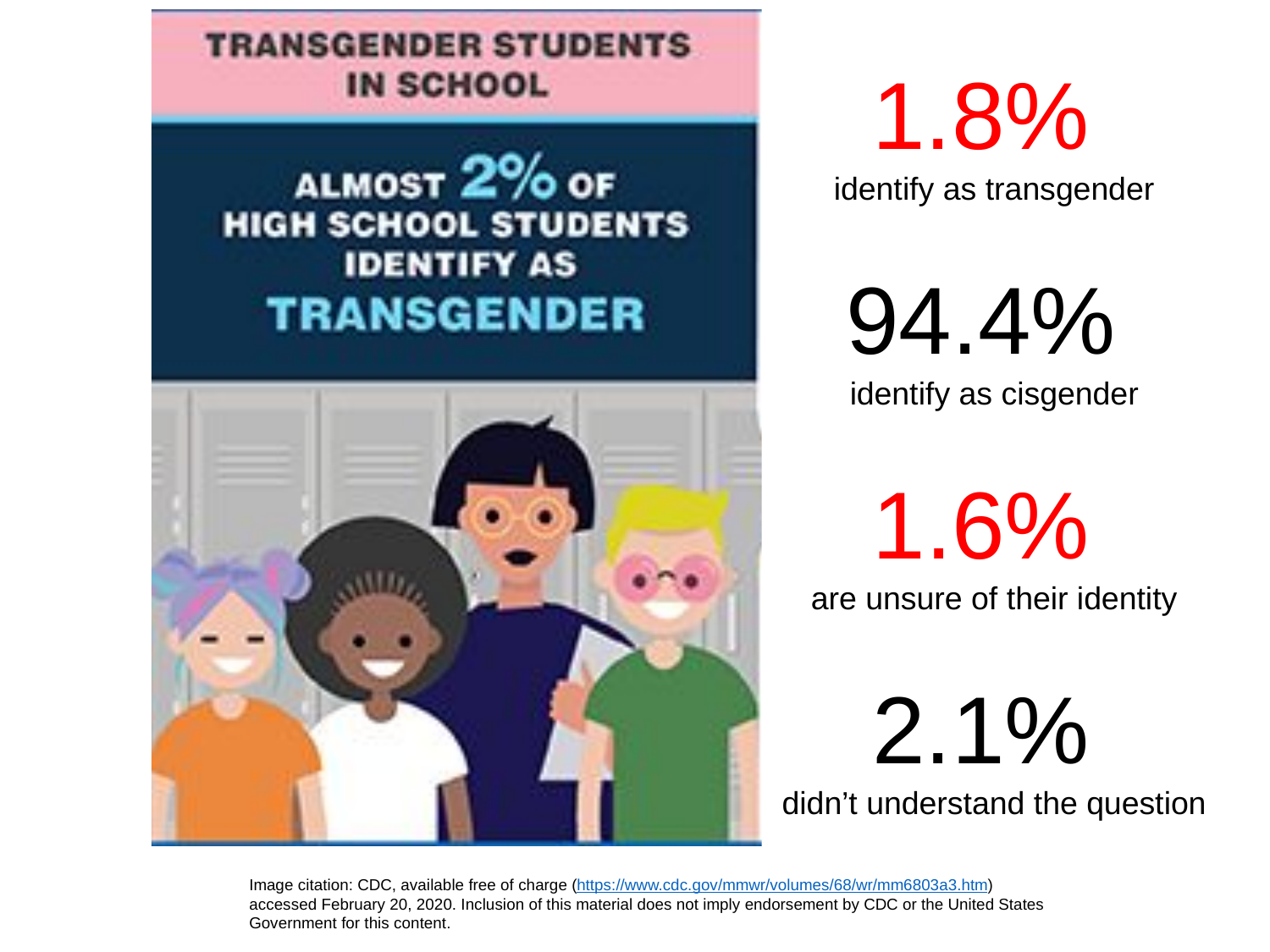

1.8%
identify as transgender
94.4%
identify as cisgender
1.6%
are unsure of their identity
2.1%
didn’t understand the question
Image citation: CDC, available free of charge (https://www.cdc.gov/mmwr/volumes/68/wr/mm6803a3.htm) accessed February 20, 2020. Inclusion of this material does not imply endorsement by CDC or the United States Government for this content.

## Slide 15
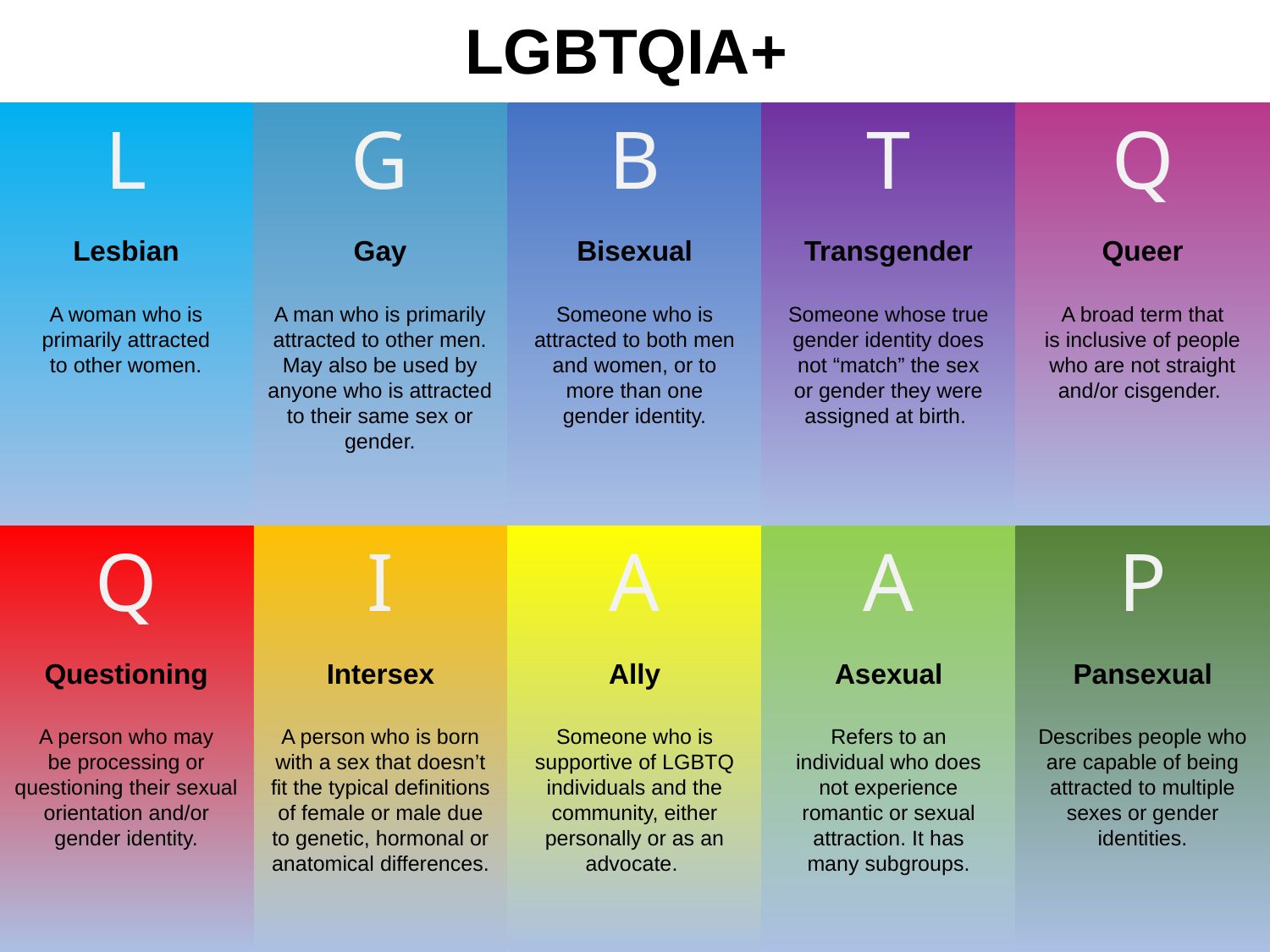

# LGBTQIA+
G
Gay
A man who is primarily attracted to other men. May also be used by anyone who is attracted to their same sex or gender.
L
Lesbian
A woman who is primarily attracted
to other women.
B
Bisexual
Someone who is attracted to both men and women, or to
more than one
gender identity.
T
Transgender
Someone whose true gender identity does
not “match” the sex
or gender they were assigned at birth.
Q
Queer
A broad term that
is inclusive of people who are not straight and/or cisgender.
I
Intersex
A person who is born with a sex that doesn’t fit the typical definitions of female or male due to genetic, hormonal or anatomical differences.
Q
Questioning
A person who may
be processing or questioning their sexual orientation and/or gender identity.
A
Ally
Someone who is supportive of LGBTQ individuals and the community, either personally or as an advocate.
A
Asexual
Refers to an
individual who does
not experience
romantic or sexual attraction. It has
many subgroups.
P
Pansexual
Describes people who are capable of being attracted to multiple sexes or gender identities.

## Slide 16
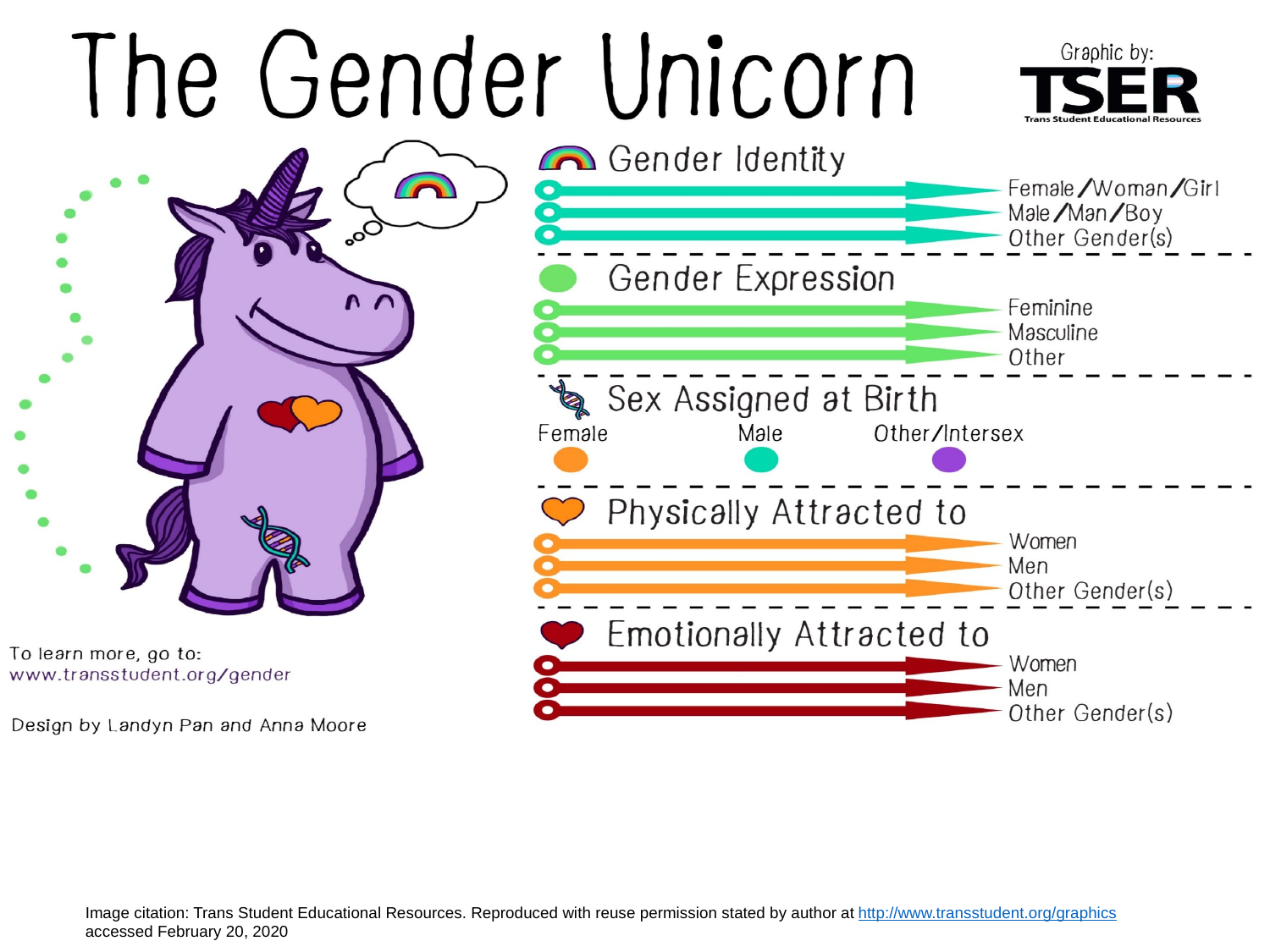

Image citation: Trans Student Educational Resources. Reproduced with reuse permission stated by author at http://www.transstudent.org/graphics accessed February 20, 2020

## Slide 17
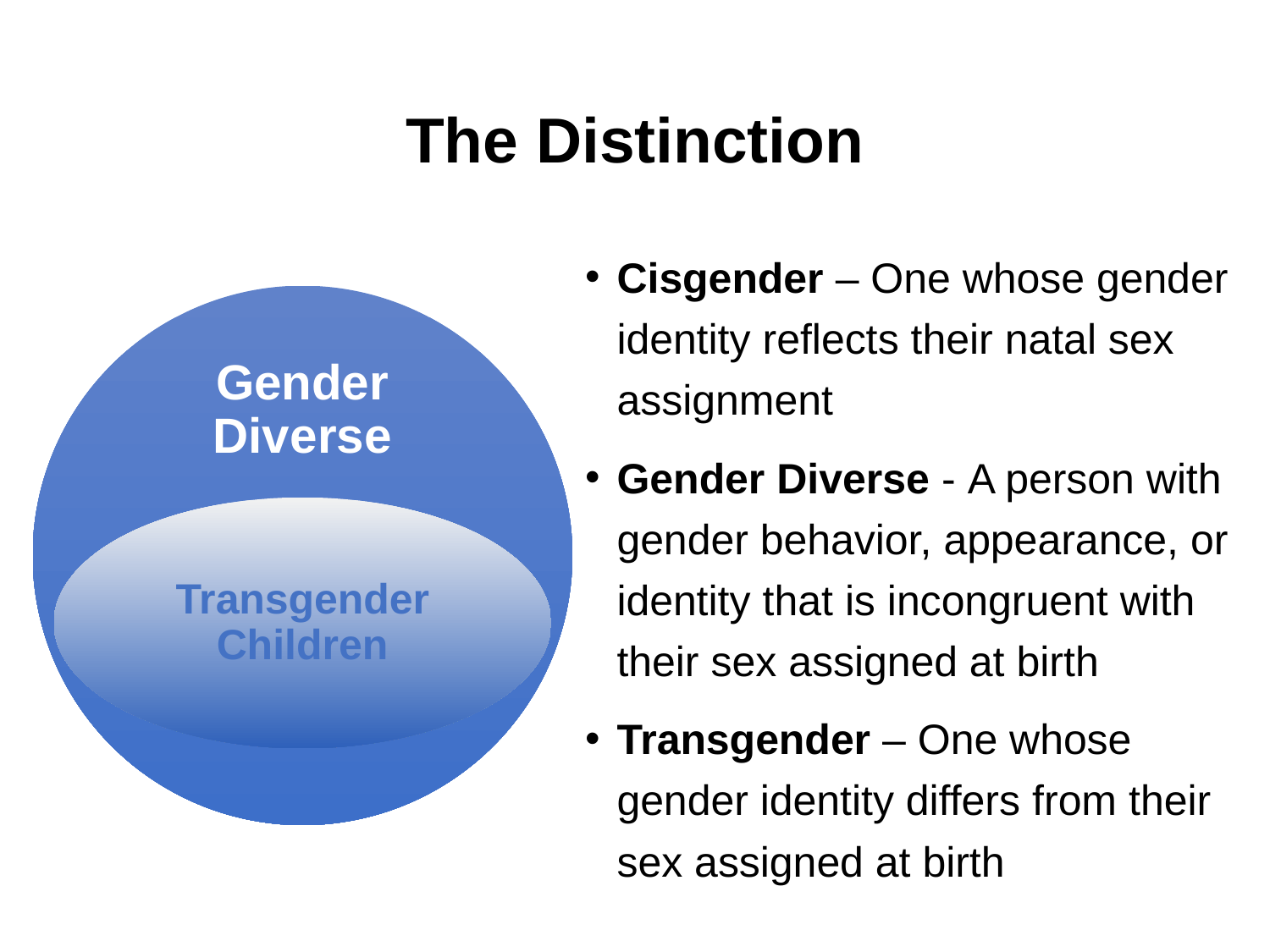

# The Distinction
Cisgender – One whose gender identity reflects their natal sex assignment
Gender Diverse - A person with gender behavior, appearance, or identity that is incongruent with their sex assigned at birth
Transgender – One whose gender identity differs from their sex assigned at birth

## Slide 18
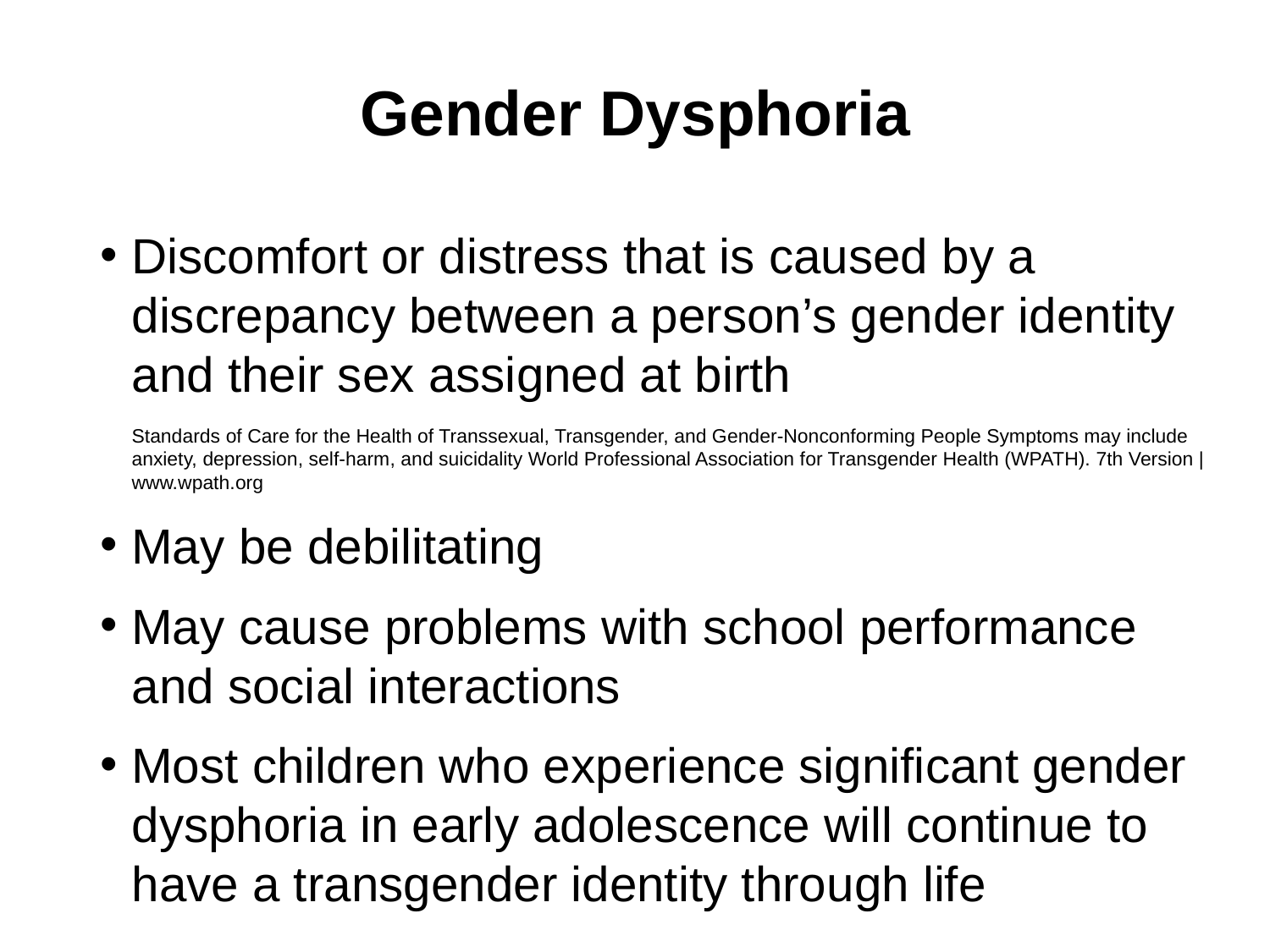

# Gender Dysphoria
Discomfort or distress that is caused by a discrepancy between a person’s gender identity and their sex assigned at birth
	Standards of Care for the Health of Transsexual, Transgender, and Gender-Nonconforming People Symptoms may include anxiety, depression, self-harm, and suicidality World Professional Association for Transgender Health (WPATH). 7th Version | www.wpath.org
May be debilitating
May cause problems with school performance and social interactions
Most children who experience significant gender dysphoria in early adolescence will continue to have a transgender identity through life

## Slide 19
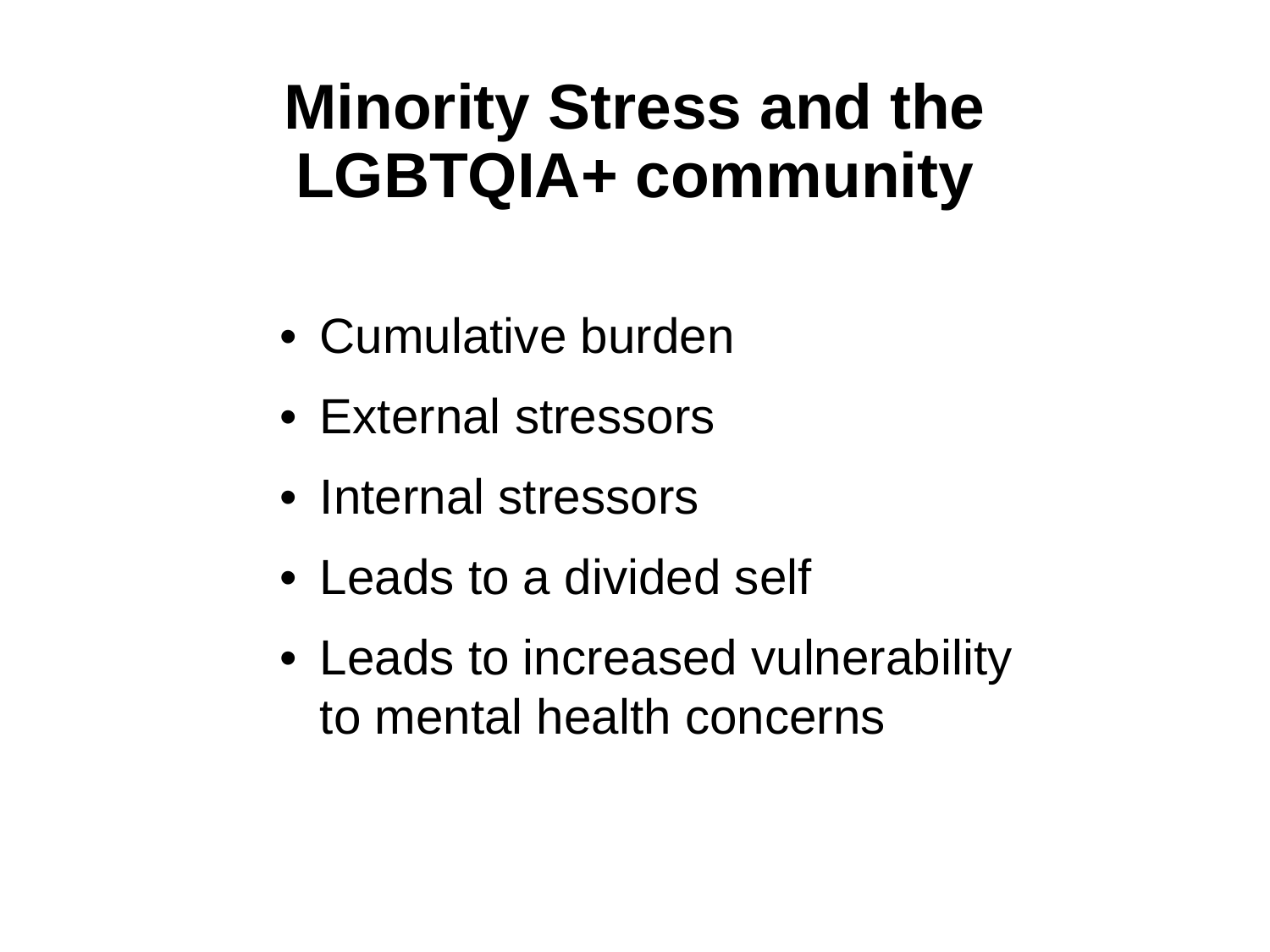

# Minority Stress and the LGBTQIA+ community
•	Cumulative burden
•	External stressors
•	Internal stressors
•	Leads to a divided self
•	Leads to increased vulnerability to mental health concerns

## Slide 20
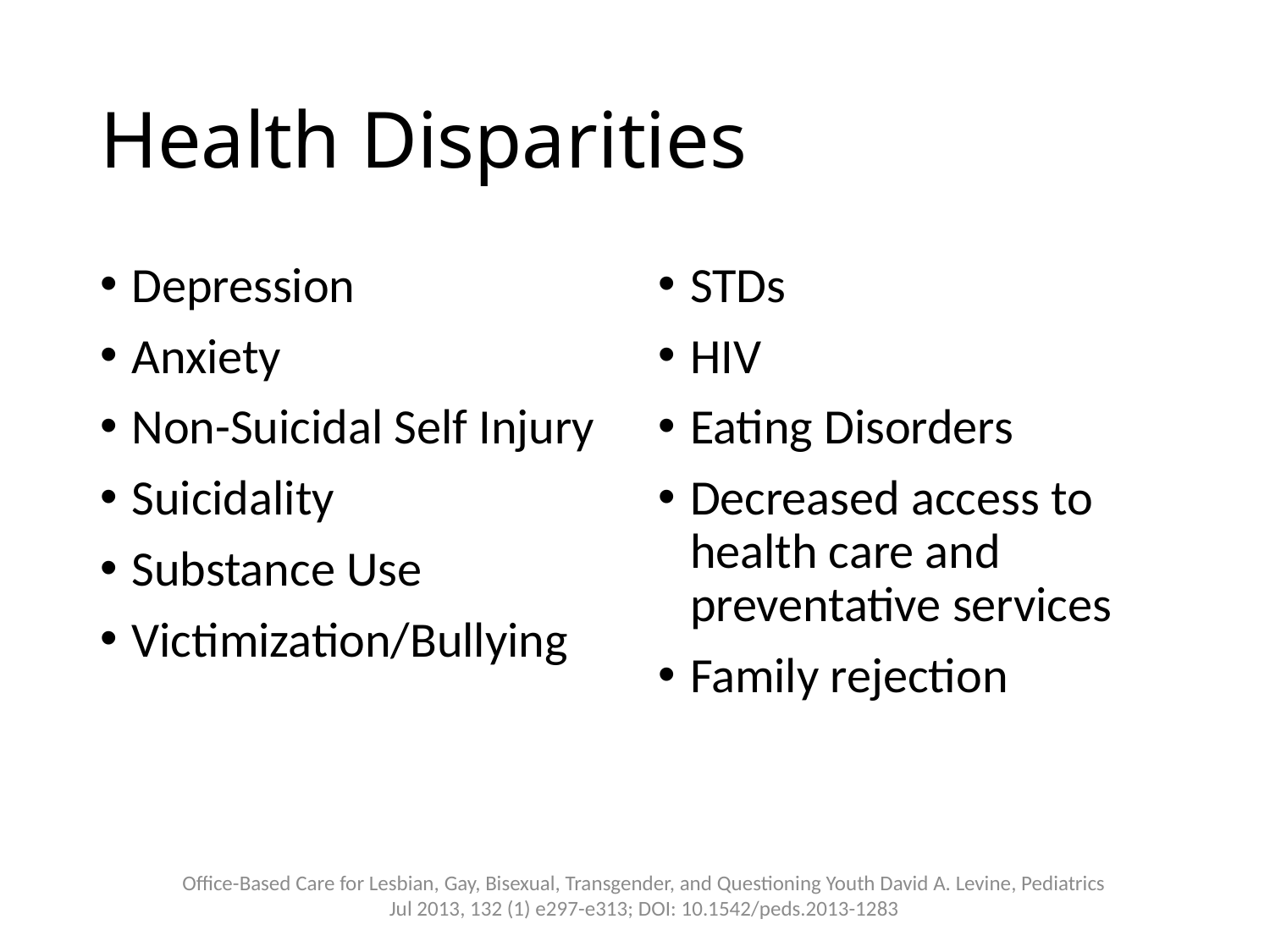

# Health Disparities
Depression
Anxiety
Non-Suicidal Self Injury
Suicidality
Substance Use
Victimization/Bullying
STDs
HIV
Eating Disorders
Decreased access to health care and preventative services
Family rejection
Office-Based Care for Lesbian, Gay, Bisexual, Transgender, and Questioning Youth David A. Levine, Pediatrics Jul 2013, 132 (1) e297-e313; DOI: 10.1542/peds.2013-1283

## Slide 21
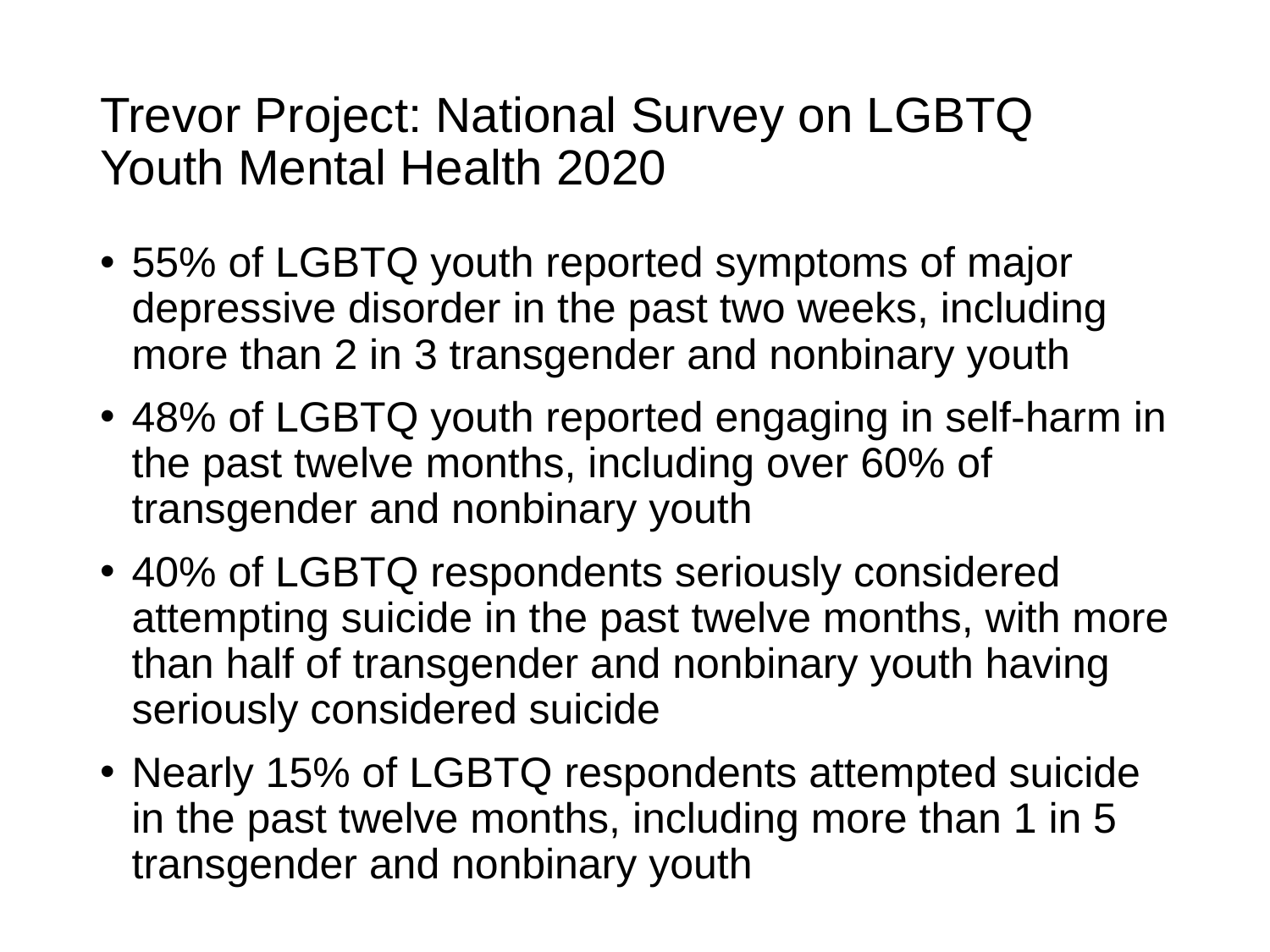

# Trevor Project: National Survey on LGBTQ Youth Mental Health 2020
55% of LGBTQ youth reported symptoms of major depressive disorder in the past two weeks, including more than 2 in 3 transgender and nonbinary youth
48% of LGBTQ youth reported engaging in self-harm in the past twelve months, including over 60% of transgender and nonbinary youth
40% of LGBTQ respondents seriously considered attempting suicide in the past twelve months, with more than half of transgender and nonbinary youth having seriously considered suicide
Nearly 15% of LGBTQ respondents attempted suicide in the past twelve months, including more than 1 in 5 transgender and nonbinary youth

## Slide 22
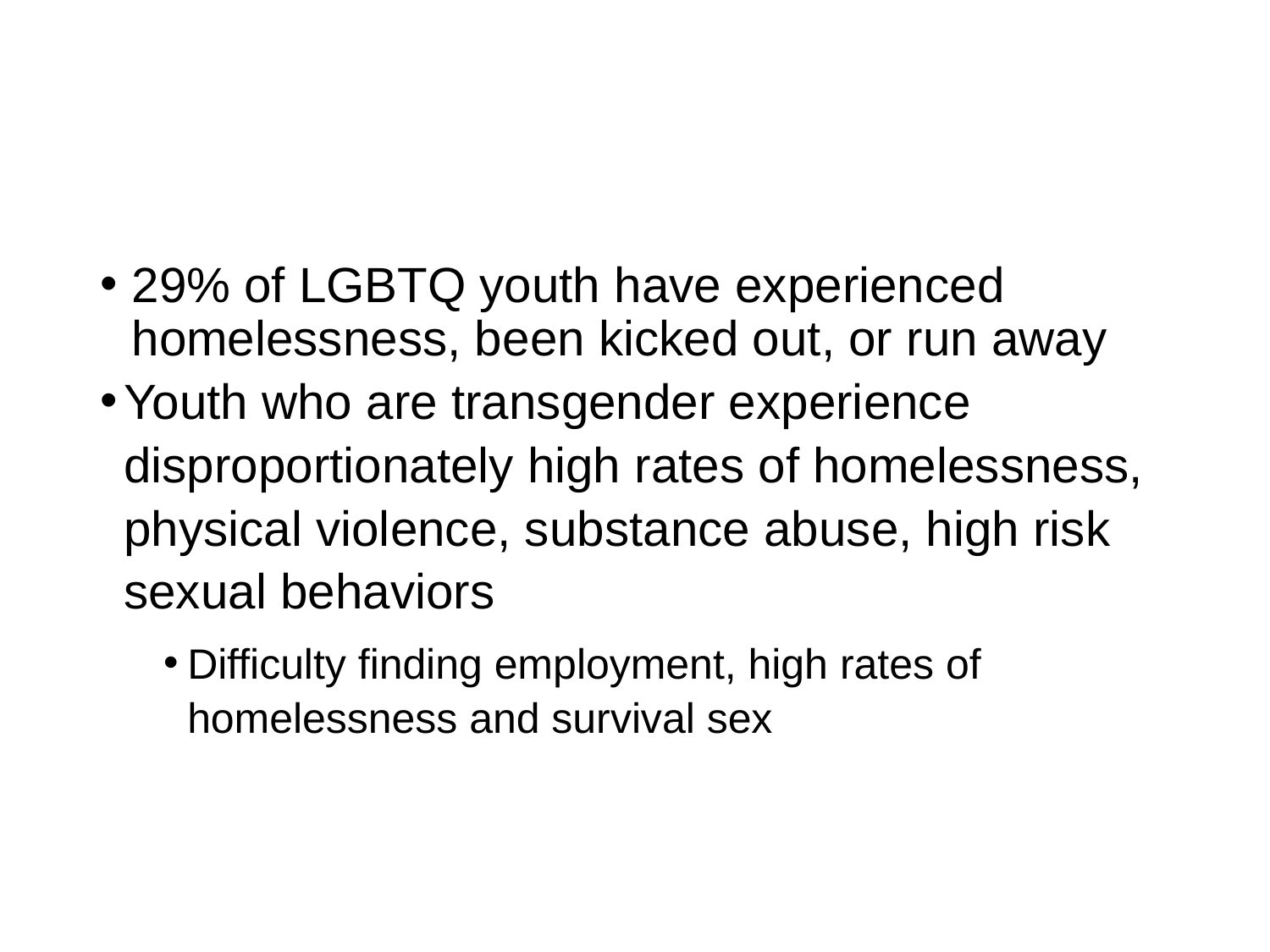

#
29% of LGBTQ youth have experienced homelessness, been kicked out, or run away
Youth who are transgender experience disproportionately high rates of homelessness, physical violence, substance abuse, high risk sexual behaviors
Difficulty finding employment, high rates of homelessness and survival sex

## Slide 23
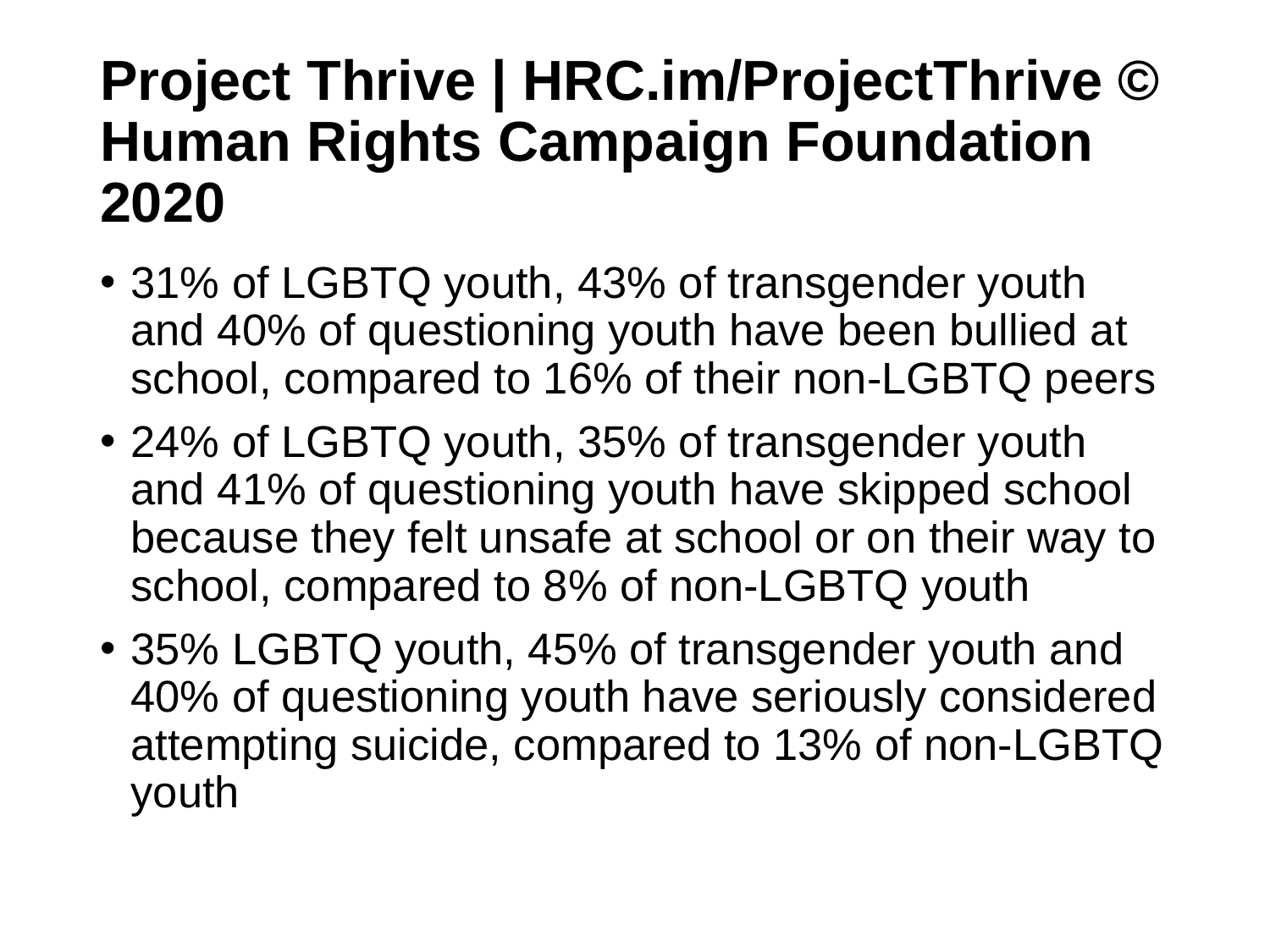

# Project Thrive | HRC.im/ProjectThrive © Human Rights Campaign Foundation 2020
31% of LGBTQ youth, 43% of transgender youth and 40% of questioning youth have been bullied at school, compared to 16% of their non-LGBTQ peers
24% of LGBTQ youth, 35% of transgender youth and 41% of questioning youth have skipped school because they felt unsafe at school or on their way to school, compared to 8% of non-LGBTQ youth
35% LGBTQ youth, 45% of transgender youth and 40% of questioning youth have seriously considered attempting suicide, compared to 13% of non-LGBTQ youth

## Slide 24
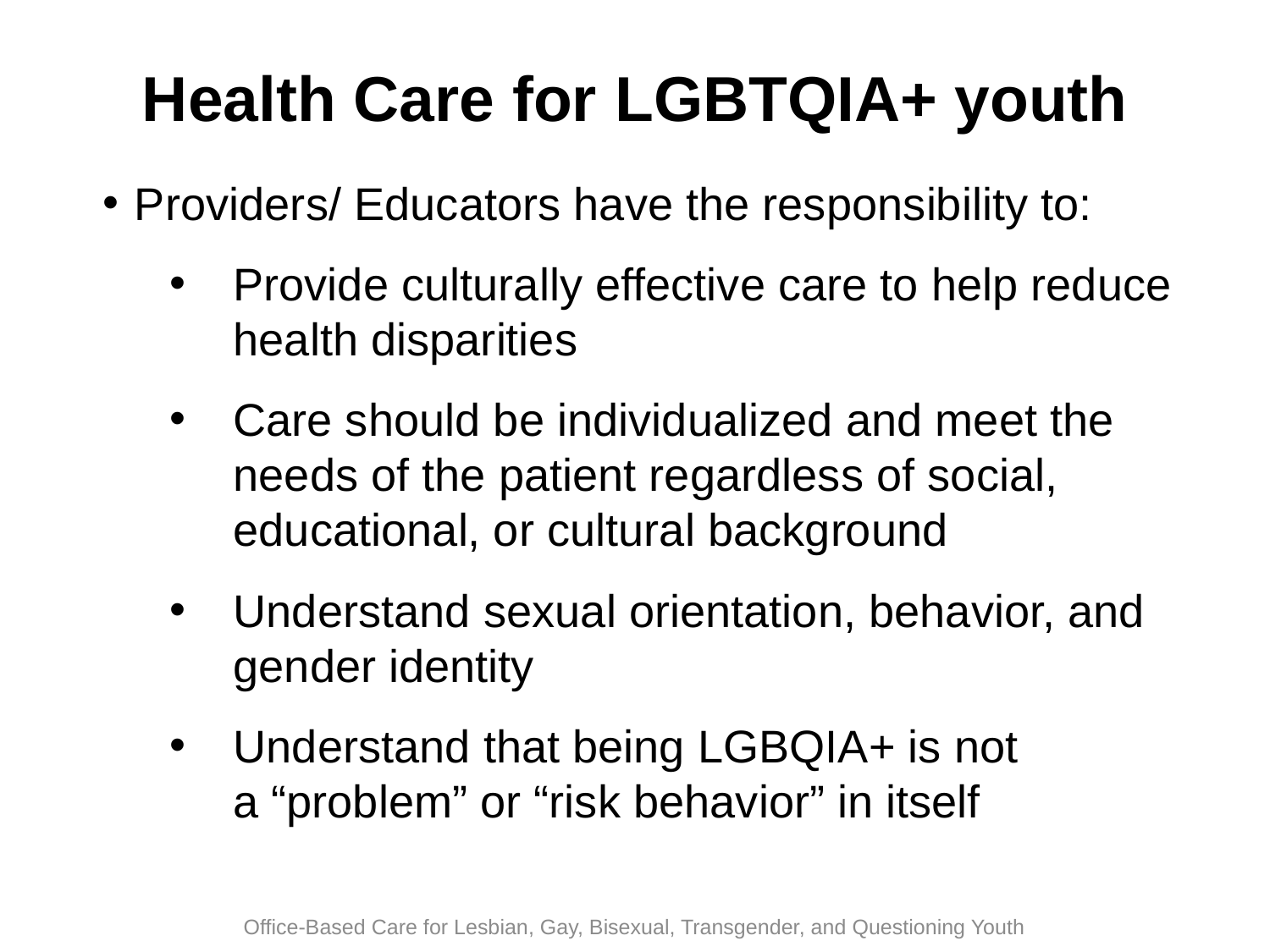

# Health Care for LGBTQIA+ youth
Providers/ Educators have the responsibility to:
Provide culturally effective care to help reduce health disparities
Care should be individualized and meet the needs of the patient regardless of social, educational, or cultural background
Understand sexual orientation, behavior, and gender identity
Understand that being LGBQIA+ is not
a “problem” or “risk behavior” in itself
Office-Based Care for Lesbian, Gay, Bisexual, Transgender, and Questioning Youth

## Slide 25
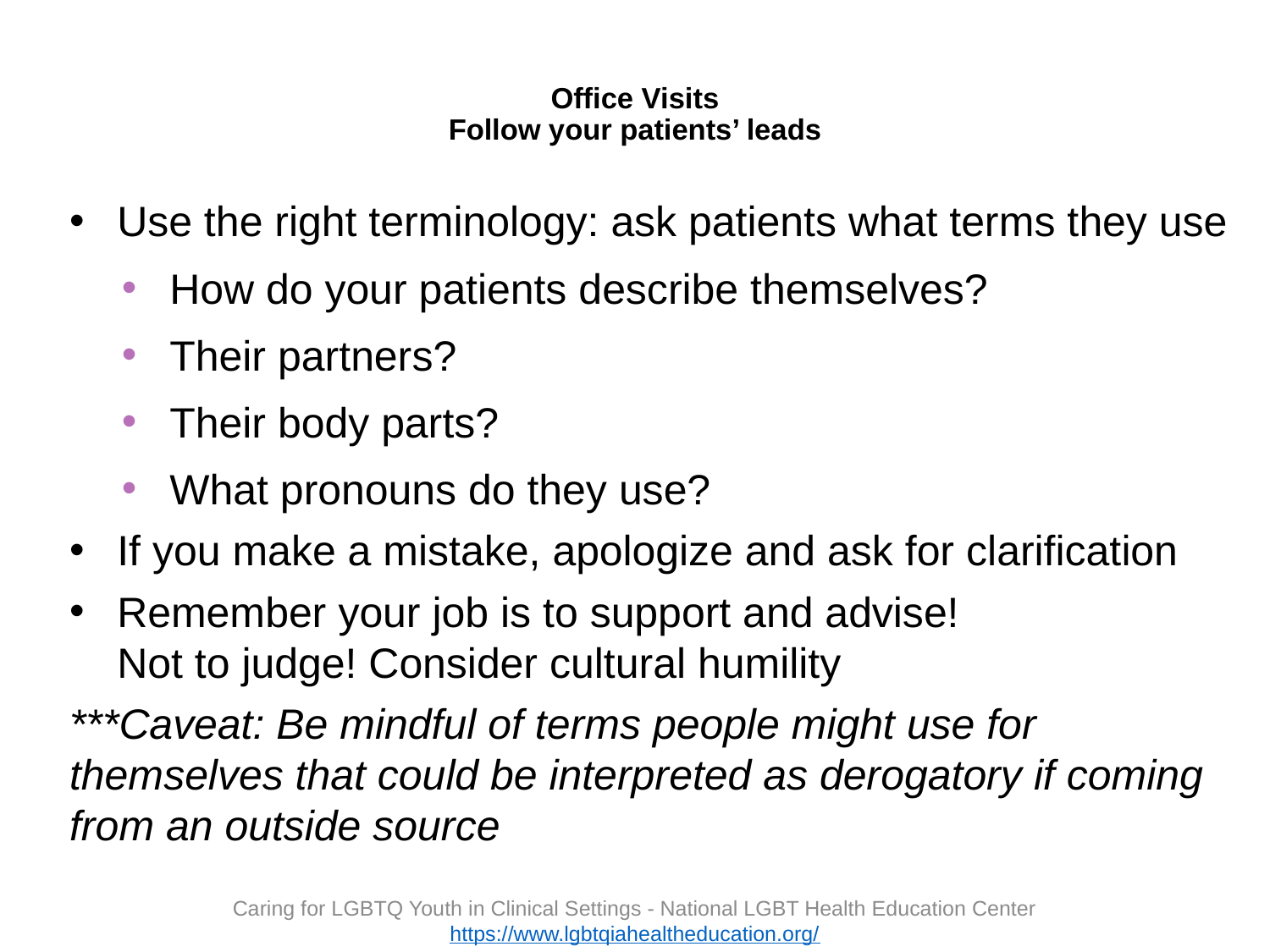

# Office Visits Follow your patients’ leads
Use the right terminology: ask patients what terms they use
How do your patients describe themselves?
Their partners?
Their body parts?
What pronouns do they use?
If you make a mistake, apologize and ask for clarification
Remember your job is to support and advise!
Not to judge! Consider cultural humility
***Caveat: Be mindful of terms people might use for themselves that could be interpreted as derogatory if coming from an outside source
Caring for LGBTQ Youth in Clinical Settings - National LGBT Health Education Center
https://www.lgbtqiahealtheducation.org/

## Slide 26
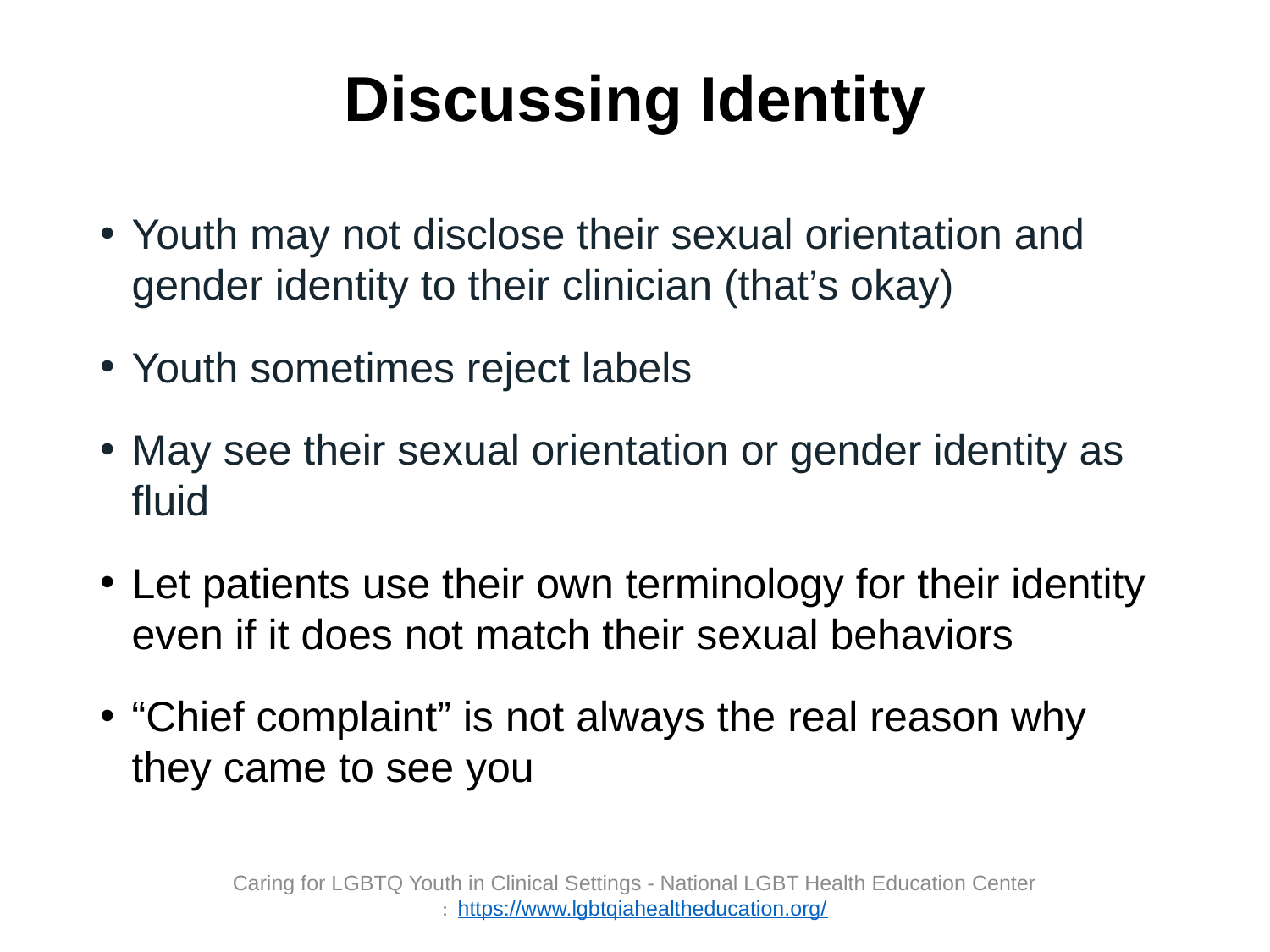

# Discussing Identity
Youth may not disclose their sexual orientation and gender identity to their clinician (that’s okay)
Youth sometimes reject labels
May see their sexual orientation or gender identity as fluid
Let patients use their own terminology for their identity even if it does not match their sexual behaviors
“Chief complaint” is not always the real reason why they came to see you
Caring for LGBTQ Youth in Clinical Settings - National LGBT Health Education Center
: https://www.lgbtqiahealtheducation.org/

## Slide 27
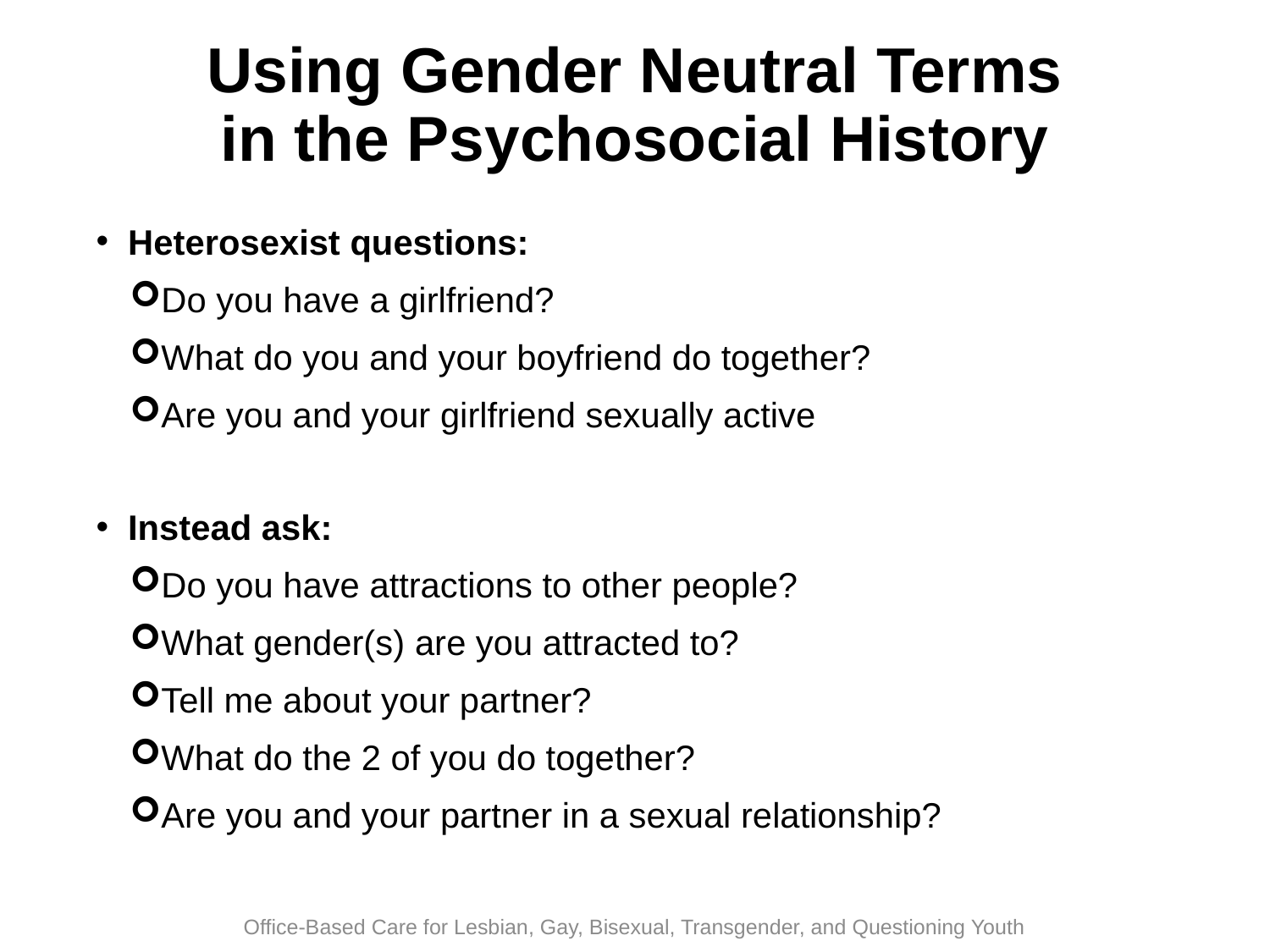

# Using Gender Neutral Terms in the Psychosocial History
Heterosexist questions:
Do you have a girlfriend?
What do you and your boyfriend do together?
Are you and your girlfriend sexually active
Instead ask:
Do you have attractions to other people?
What gender(s) are you attracted to?
Tell me about your partner?
What do the 2 of you do together?
Are you and your partner in a sexual relationship?
Office-Based Care for Lesbian, Gay, Bisexual, Transgender, and Questioning Youth

## Slide 28
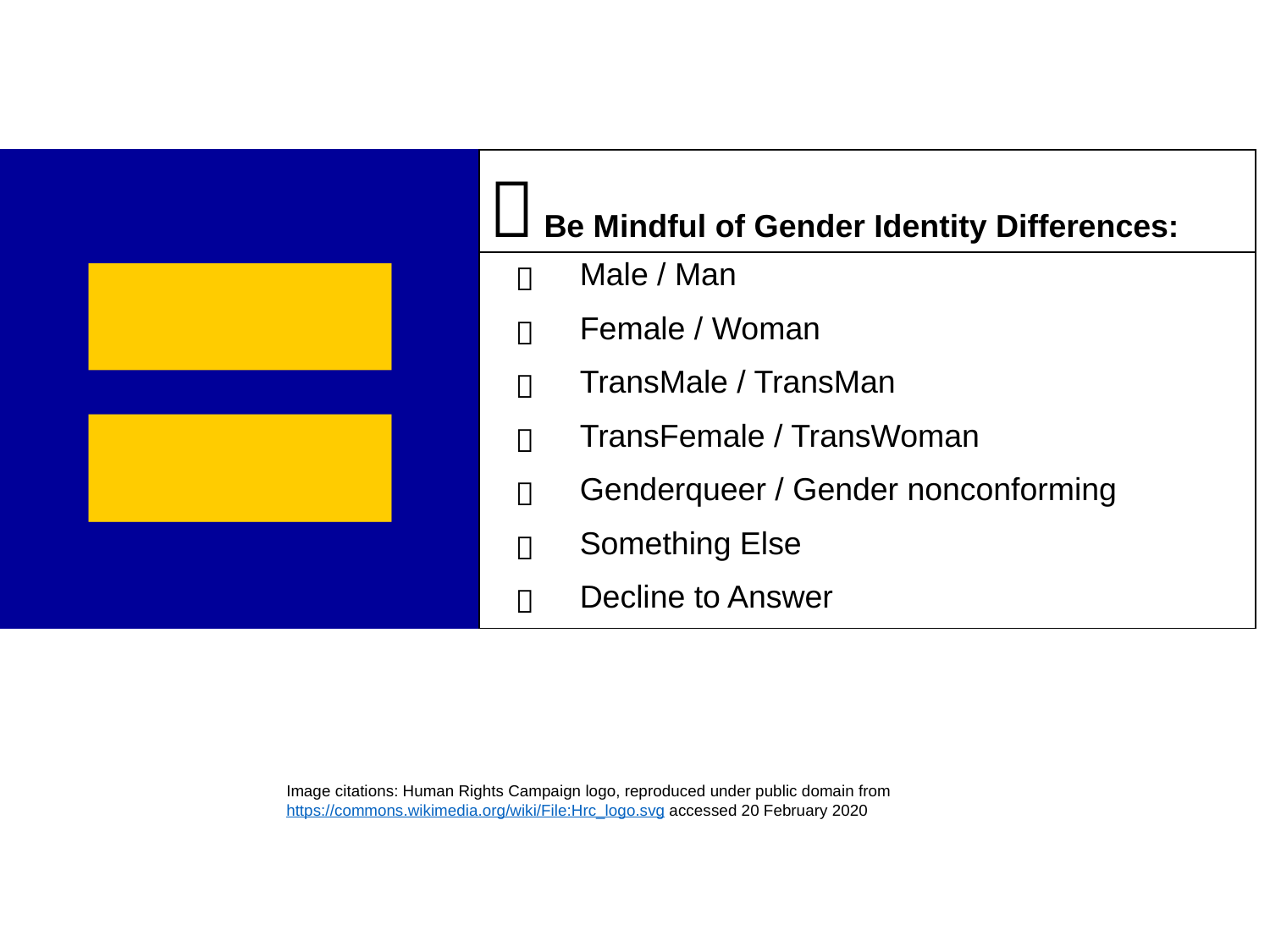

| α Be Mindful of Gender Identity Differences: | |
| --- | --- |
|  | Male / Man |
|  | Female / Woman |
|  | TransMale / TransMan |
|  | TransFemale / TransWoman |
|  | Genderqueer / Gender nonconforming |
|  | Something Else |
|  | Decline to Answer |
Image citations: Human Rights Campaign logo, reproduced under public domain from
https://commons.wikimedia.org/wiki/File:Hrc_logo.svg accessed 20 February 2020

## Slide 29
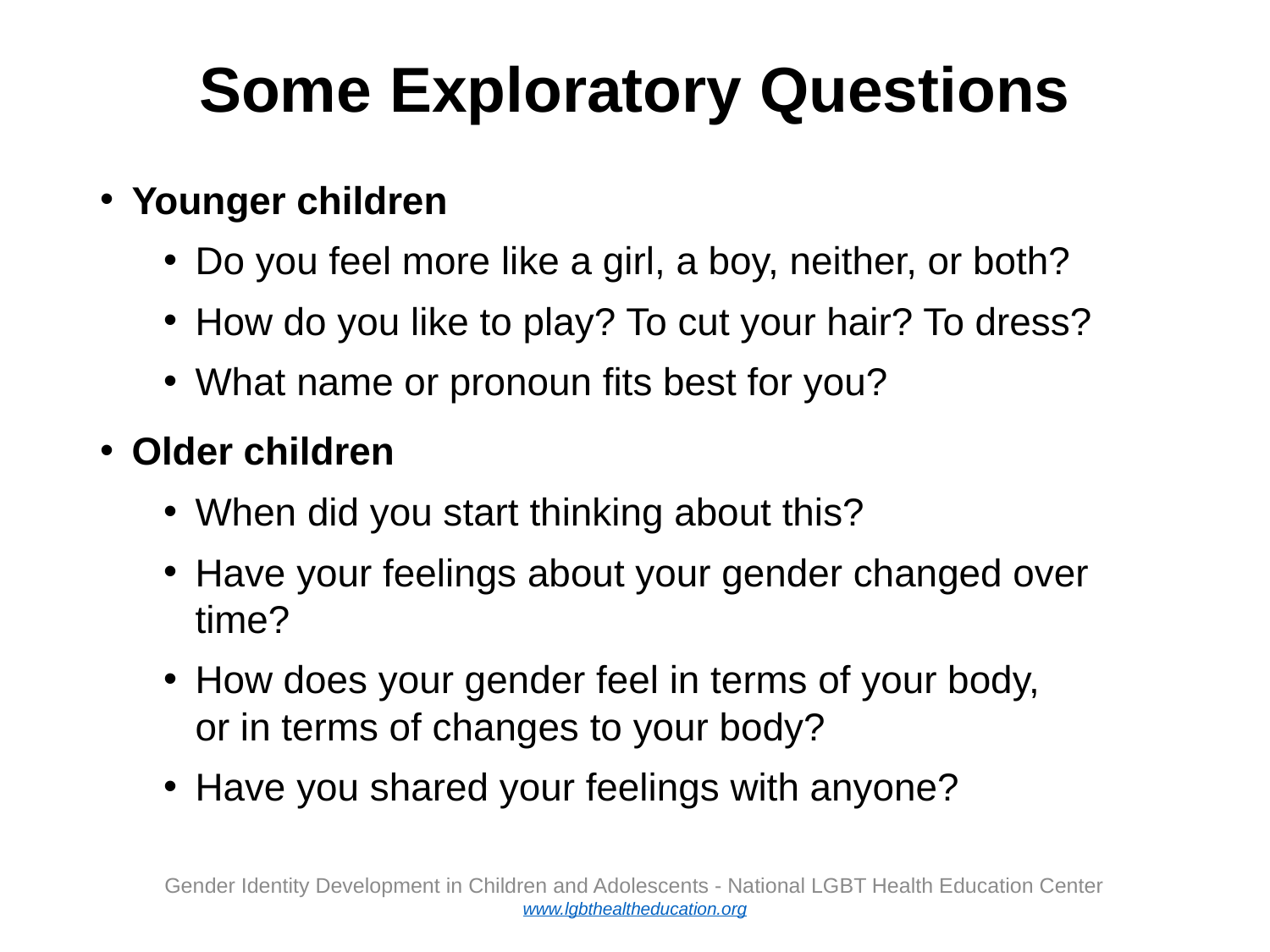

# Some Exploratory Questions
Younger children
Do you feel more like a girl, a boy, neither, or both?
How do you like to play? To cut your hair? To dress?
What name or pronoun fits best for you?
Older children
When did you start thinking about this?
Have your feelings about your gender changed over time?
How does your gender feel in terms of your body,
or in terms of changes to your body?
Have you shared your feelings with anyone?
Gender Identity Development in Children and Adolescents - National LGBT Health Education Center
www.lgbthealtheducation.org

## Slide 30
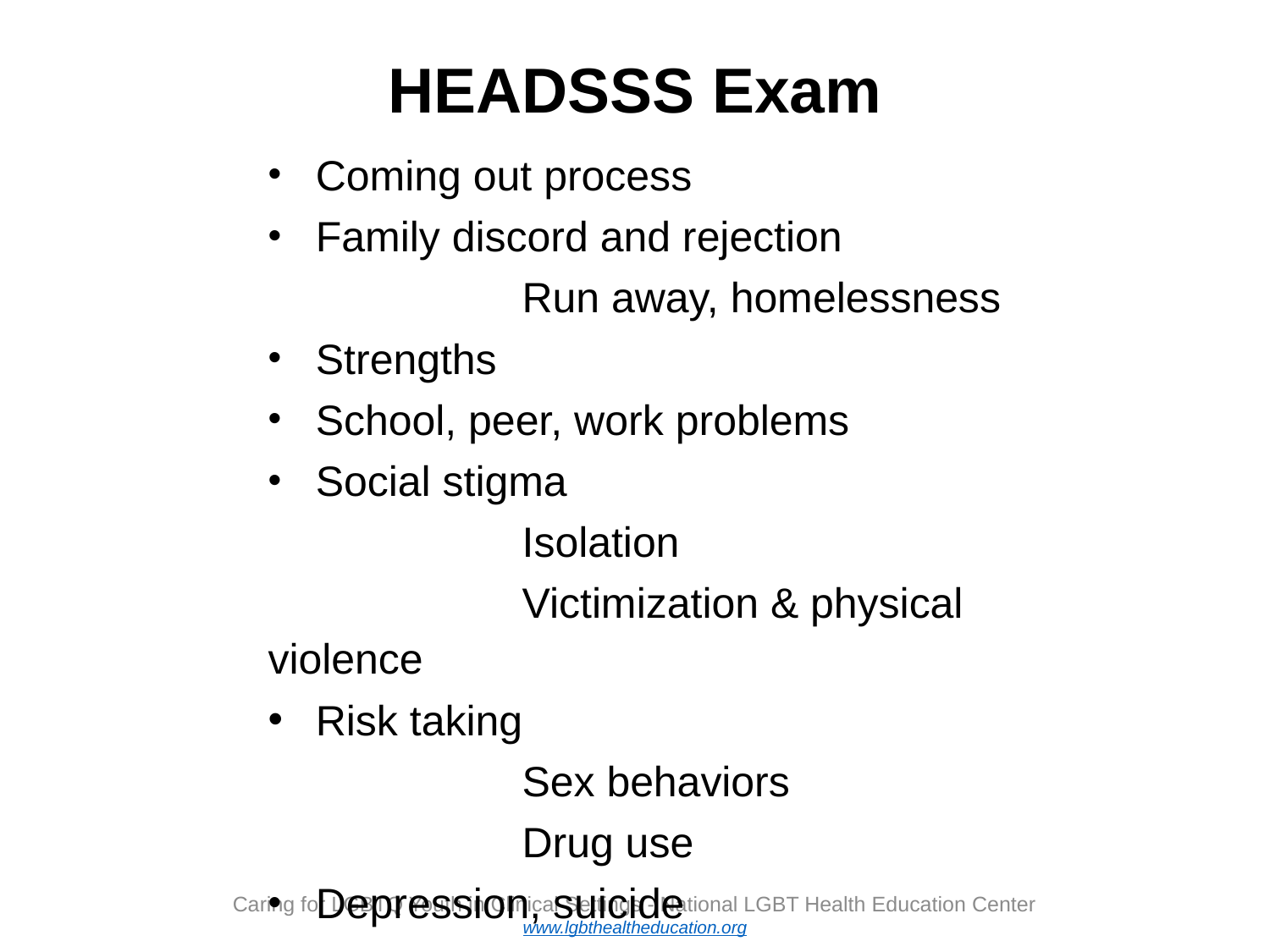

# HEADSSS Exam
Coming out process
Family discord and rejection
		Run away, homelessness
Strengths
School, peer, work problems
Social stigma
		Isolation
		Victimization & physical violence
Risk taking
		Sex behaviors
		Drug use
Depression, suicide
Caring for LGBTQ Youth in Clinical Settings - National LGBT Health Education Center
www.lgbthealtheducation.org

## Slide 31
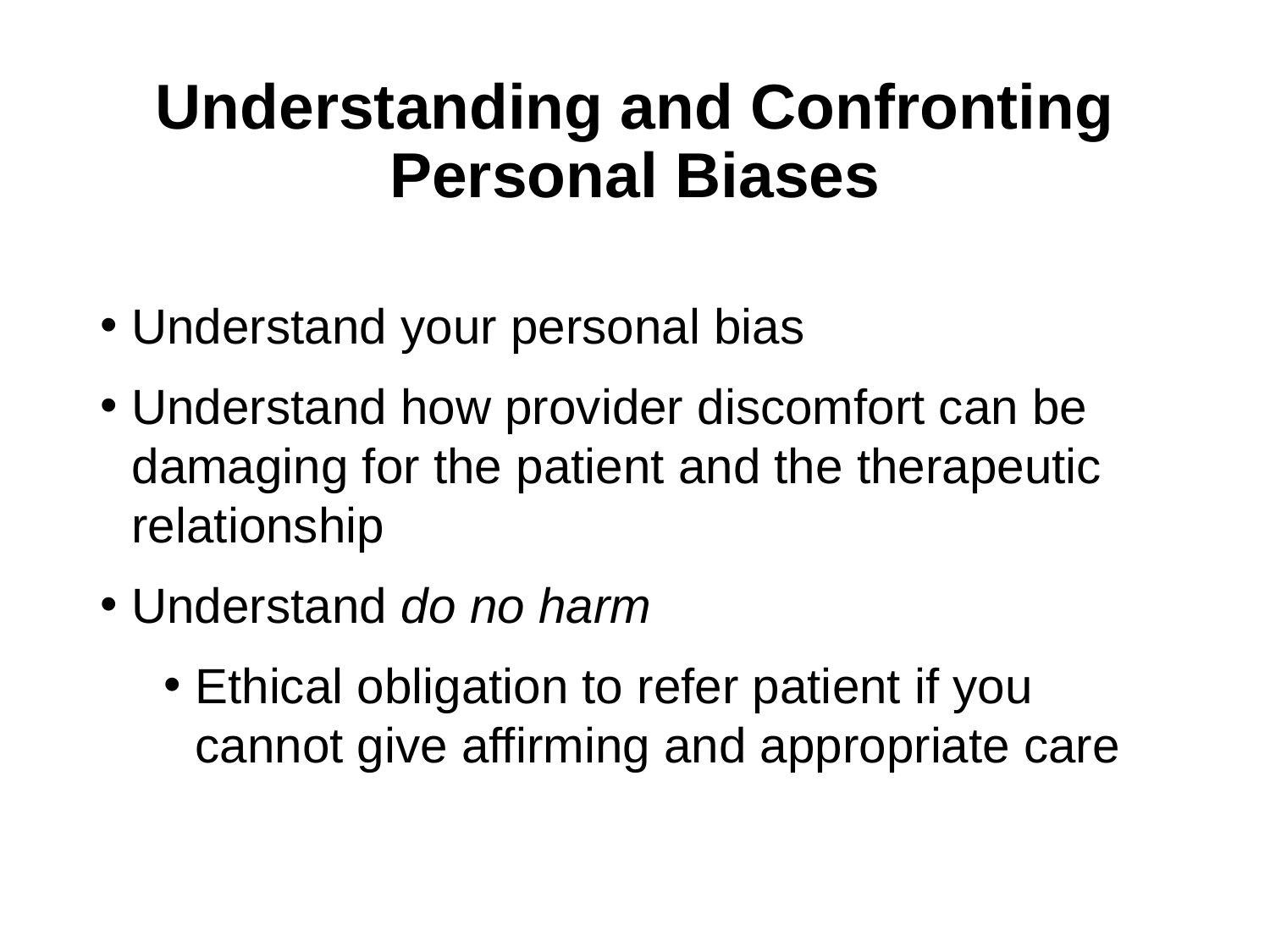

# Understanding and Confronting Personal Biases
Understand your personal bias
Understand how provider discomfort can be damaging for the patient and the therapeutic relationship
Understand do no harm
Ethical obligation to refer patient if you cannot give affirming and appropriate care

## Slide 32
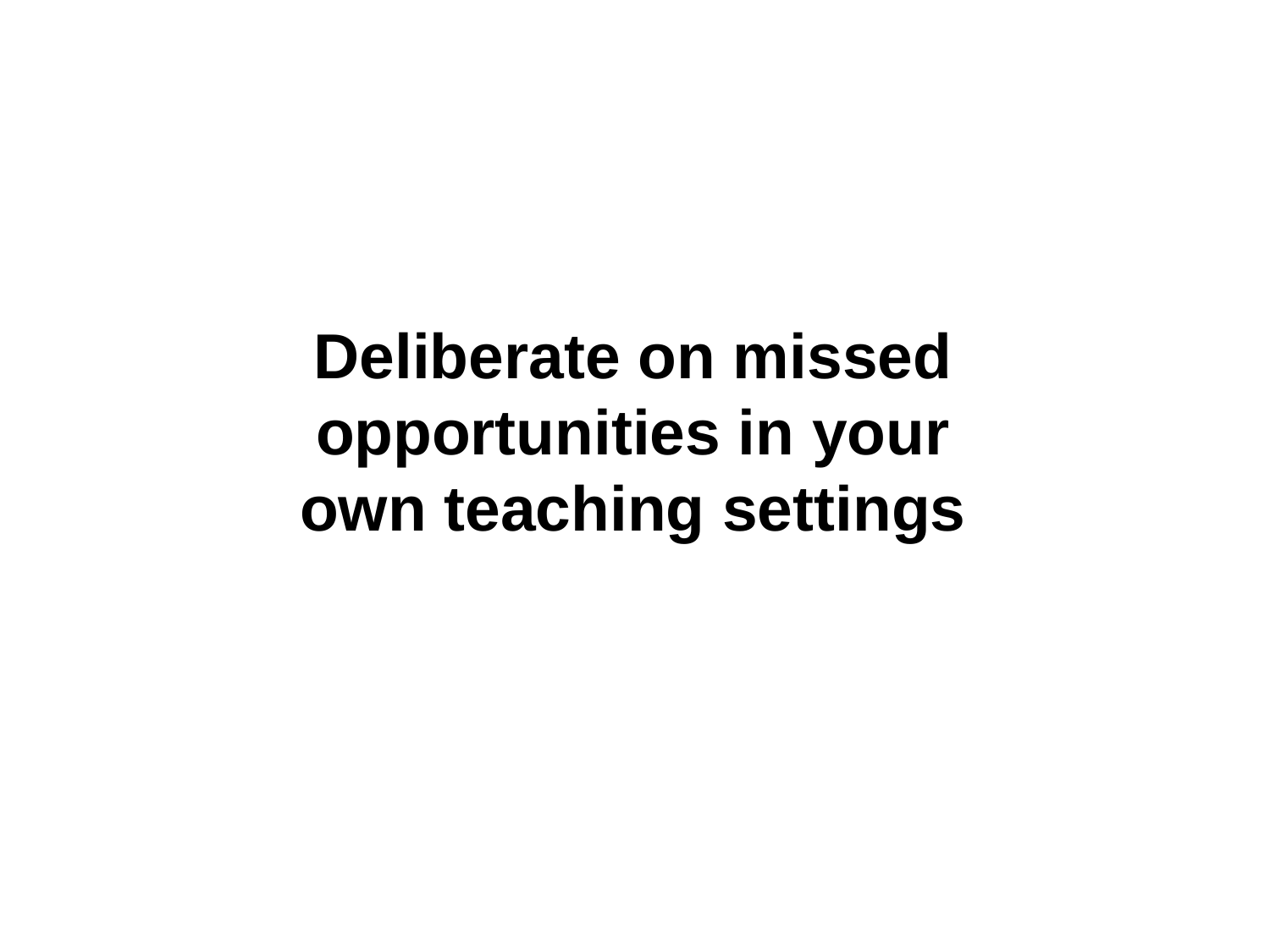

# Deliberate on missed opportunities in your own teaching settings
