# Supplementary material for: Teaching Sexual Orientation and Gender Identity in Pediatric Clinical Settings: A Training Workshop for Faculty and Residents
Source: MedEdPORTAL. 2021 Apr 5;17:11137. doi: 10.15766/mep_2374-8265.11137 (PMC8034234; doi:10.15766/mep_2374-8265.11137)
Supplement: Supplementary file 1 — Facilitator Guide.docxPatient Vignettes.pptxDidactic Presentation.pptxSelected Educational Resources.docxCase Discussion with Role-Play Opportunities.docxEvaluation Form.docx [file mep_2374-8265.11137-s001.zip › D. Selected Educational Resources.docx]

# Appendix D

## Selected Educational Resources and Materials


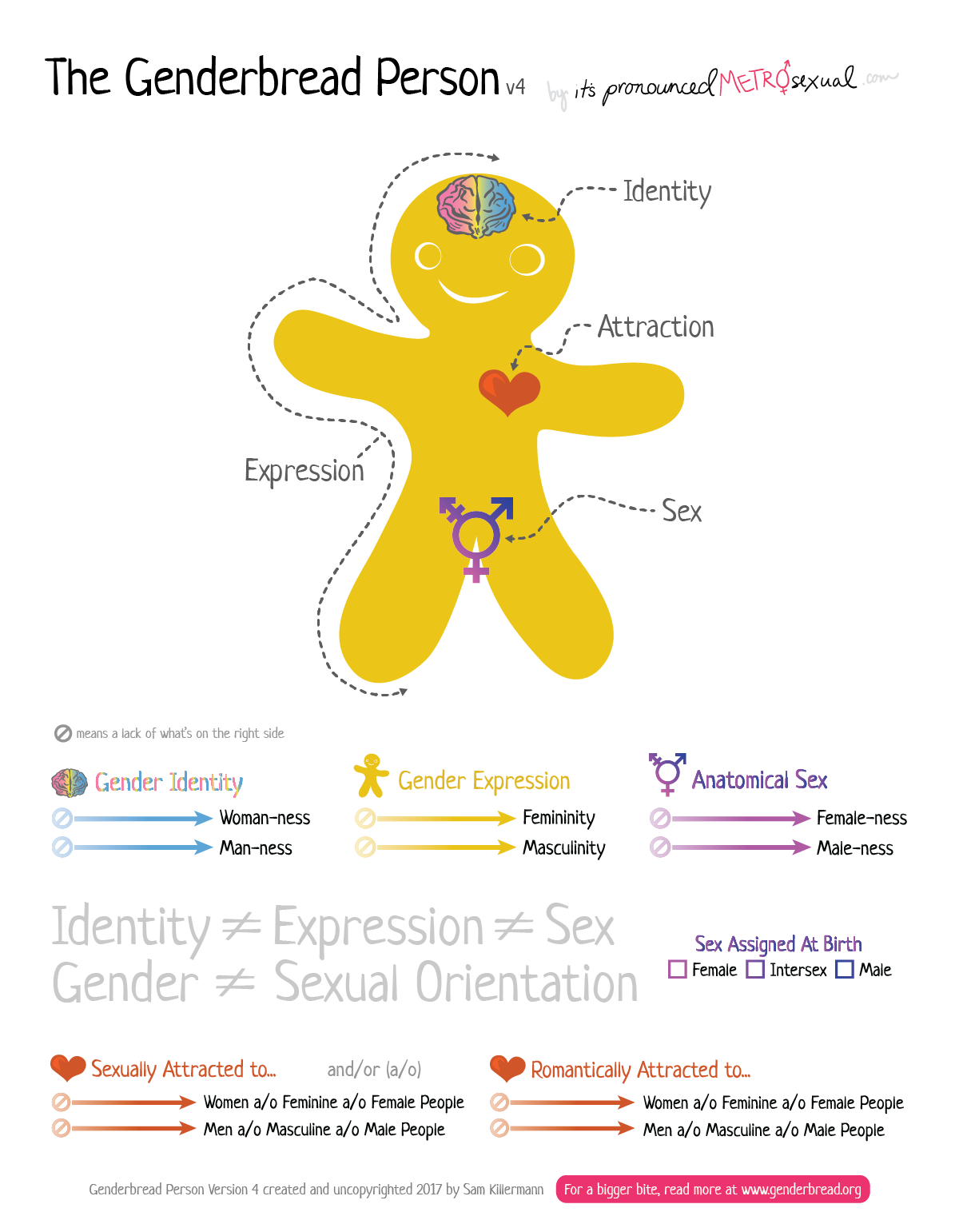


**GLOSSARY. Aiming for culturally-humble care, we recognize: *As we learn more, our language can change.***

**These sites keep up-to-date with the most appropriate terminology.**

1. The Trevor Project. <https://www.thetrevorproject.org/trvr_support_center/glossary/>
2. Safe Zone Project. LBGBTQ+ Vocabulary Glossary of Terms. <https://thesafezoneproject.com/resources/vocabulary/>
3. Amnesty International. LGBT Glossary. <https://www.amnestyusa.org/pdfs/toolkit_LGBTglossary.pdf>
4. PFLAG National Glossary of Terms. <https://pflag.org/glossary>
5. Human Rights Campaign. Glossary of Terms. <https://www.hrc.org/resources/glossary-of-terms>
6. Lambda Legal Glossary of LGBTQ Terms <https://www.lambdalegal.org/know-your-rights/article/youth-glossary-lgbtq-terms?gclid=EAIaIQobChMI2YuC0feX6AIVxsDACh3N_w9yEAAYASAAEgKj2fD_BwE>

**GENERAL REFERENCES.**

1. AAP Section on LGBT Health & Wellness. Supporting and Caring for Transgender Children. 2016; <http://hrc.im/supportingtranschildren>.
2. The Genderbread Person. Version 3:[https://www.itspronouncedmetrosexual.com/2015/03/the-genderbread-person-v3/.](https://www.itspronouncedmetrosexual.com/2015/03/the-genderbread-person-v3/)
3. Shumer DE, Nokoff NJ, Spack NP. Advances in the Care of Transgender Children and Adolescents. *Adv Pediatr.* 2016;63(1):79-102.
4. Olson-Kennedy J, Rosenthal SM, Hastings J, Wesp L. Health considerations for gender non-conforming children and transgender adolescents. 2016; <https://transcare.ucsf.edu/guidelines/youth>.
5. Mahfouda S, Moore JK, Siafarikas A, Zepf FD, Lin A. Puberty suppression in transgender children and adolescents. *The Lancet Diabetes & Endocrinology.* 2017;5(10):816-826.
6. Grant JM, Mottet LA, Tanis J. *National Transgender Discrimination Survey Report on health and health care.* Washington, D.C.: National Center for Transgender Equality & National Gay and Lesbian Task Force; 2010.
7. Sex and Gender Health Collaborative. Health Is Affected by Both Sex and Gender. <https://www.amwa-doc.org/sghc/>
8. Sex and Gender Specific Health; [https://www.sexandgenderhealth.org/.](https://www.sexandgenderhealth.org/)
9. The Fenway Institute [www.lgbtqiahealtheducation.org](http://www.lgbtqiahealtheducation.org)
10. Standards of Care for the Health of Transsexual, Transgender, and Gender-Nonconforming People. World Professional Association for Transgender Health (WPATH) 2012; <https://www.wpath.org/publications/soc>

**SPECIALTY REFERENCES.**

1. Cipres D, Seidman D, Cloniger C, 3rd, Nova C, O'Shea A, Obedin-Maliver J. Contraceptive use and pregnancy intentions among transgender men presenting to a clinic for sex workers and their families in San Francisco. *Contraception.* 2017;95(2):186-189.
2. Light AD, Obedin-Maliver J, Sevelius JM, Kerns JL. Transgender men who experienced pregnancy after female-to-male gender transitioning. *Obstetrics and gynecology.* 2014;124(6):1120-1127.
3. *FSRH CEU Statement: Contraceptive Choices and Sexual Health for Transgender and Non-binary People.* Edinburgh, Scotland, UK: The Faculty of Sexual & Reproductive Healthcare of the Royal college of Obststricians and Gynaecologists;2017.
4. National Center for HIV/AIDS, Viral Hepatitis, STD, and TB Prevention, Division of HIV/AIDS Prevention. Pre-exposure Prophylaxis (PrEP) for HIV Prevention. 2014; <https://www.cdc.gov/nchhstp/newsroom/docs/factsheets/prep-factsheet-508.pdf>
5. Hosek SG, Landovitz RJ, Kapogiannis B, et al. Safety and Feasibility of Antiretroviral Preexposure Prophylaxis for Adolescent Men Who Have Sex With Men Aged 15 to 17 Years in the United States. *JAMA pediatrics.* 2017;171(11):1063-1071.
6. Vlot MC, Klink DT, den Heijer M, Blankenstein MA, Rotteveel J, Heijboer AC. Effect of pubertal suppression and cross-sex hormone therapy on bone turnover markers and bone mineral apparent density (BMAD) in transgender adolescents. *Bone.* 2017;95:11-19.
7. Lopez CM, Solomon D, Boulware SD, Christison-Lagay ER. Trends in the use of puberty blockers among transgender children in the United States. *J Pediatr Endocrinol Metab.* 2018;31(6):665-670.
