# Supplementary material for: Teaching Sexual Orientation and Gender Identity in Pediatric Clinical Settings: A Training Workshop for Faculty and Residents
Source: MedEdPORTAL. 2021 Apr 5;17:11137. doi: 10.15766/mep_2374-8265.11137 (PMC8034234; doi:10.15766/mep_2374-8265.11137)
Supplement: Supplementary file 1 — Facilitator Guide.docxPatient Vignettes.pptxDidactic Presentation.pptxSelected Educational Resources.docxCase Discussion with Role-Play Opportunities.docxEvaluation Form.docx [file mep_2374-8265.11137-s001.zip › E. Case Discussion with Role-Play Opportunities.docx]

# Appendix E: Case Scenarios for Small Groups

## Instructions:

- Choose the case(s) that would be most appropriate for the learners; these cases are designed for small group discussion (groups up to 6-8), and for role-play in triads (groups of 3)
- Ask for a volunteer in the small group to read out the scenario & serve as reporter for a large-group debrief
- Answer the discussion questions (10 min)
- Facilitators: Key discussion points for each question are provided on the page after each case
- Optional: If time permits, role-play the scenario as instructed within each scenario (5 min)

Note: These cases do not represent the full breadth and depth of LGBTQIA+ issues such as sexually transmitted infections or contraceptive discussions. It is recommended that workshop leaders address this lack of full inclusivity at the start of the session.

## Cases:

| Pages | Scenario title | Roles for role play | Key topics for discussion |
| --- | --- | --- | --- |
| 2-4 | Case 1: “I’m concerned about this patient’s cutting behavior” | Patient  Faculty  Medical student | - Use of HEADSS exam in teaching - Psychiatric risks for transgender patients - Patient education - Teaching communication during adolescent encounter |
| 5-8 | Case 2: “I broke my arm!” | Faculty  Patient  Patient’s parent | - Abuse risks for transgender patients - Use and effects of hormonal therapies - Effective communication with transgender patients - Teaching communication during adolescent encounter - Patient/Parent/Provider Communication |
| 8-10 | Case 3: A matter of risk | Faculty  Patient’s parent  Observer | - Subspecialty engagement in care of transgender patients - Use and effects of hormonal therapies - Effective communication with transgender patients - Patient/Parent/Provider Communication |
| 11-14 | Case 4: 6-year old walk in | Patient’s parent  Faculty  Resident | - Gender expression in younger children - Effective communication with parents - Teaching and modeling proper communication skills for learners - Patient/Parent/Provider Communication - Modeling use of clinical guidelines content in direct patient care settings |

## Case 1: “I’m concerned about this patient’s cutting behavior”

*Developed by Thanakorn Jirasevijinda, M.D.*

Role: Faculty

Scenario:

You are precepting an eager medical student (M3), Madison, in continuity clinic, who’s just seen a 16-year-old patient, Lee, for his yearly physical. Madison has been practicing the HEADSS framework for gathering adolescent social history, and has found the framework helpful for opening up adolescent patients and gaining their trust.

After presenting Lee’s H&P, Madison confidently summarizes this assessment: “Lee is an otherwise healthy 16-year-old adolescent patient who presents for his yearly physical, has no complaints today, is up to date with his immunizations. However, I am concerned about his HEADSS screen, which elicited depressed mood and suicidal ideation in the past though not at this time. I also found a few linear scars on his left arm and both thighs. I’m concerned about this patient’s cutting behavior, and that he may progress to suicidal attempts and we have to intervene urgently. I would like to send him to the ED for a psych consult and more thorough evaluation for possible inpatient psychiatric treatment.”

You commend Madison for a nice job gathering detailed history using the HEADSS screening tool & for giving a focused presentation.

Discussion Questions:

1. What are some missed opportunities in Madison’s interactions with this adolescent patient?
2. How would you recommend Madison proceed with additional data gathering?
3. What plans would you recommend for Lee for this visit? What follow-up plans?
4. What resources would you recommend for Madison to learn more?

## Case 1: “I’m concerned about this patient’s cutting behavior”

### Instructor Guide with Suggested Responses

Question 1:

What are some missed opportunities in Madison’s interactions with this adolescent patient?

- - - As learners gain more skills in using the HEADSS model, they are able to elicit more information from the latter part of the HEADSS. However, one common pitfall is not delving deeper: 1) They may be satisfied with the information already gathered (suicidal ideation, etc.); 2) They may lack skills at exploring further; 3) They may be afraid of what to do with the additional information uncovered (e.g., what to do with disclosure re suicidal ideation).
    - In this case, Madison has done a nice job building trust and soliciting information about Lee’s mental health issues. The next step would be to delve deeper into the “Why?” LBGT youths engage in cutting behavior, experience depression and had suicidal thoughts at a higher rate than their non-LGBT counterparts (See slides). Delving further includes assessing sexual orientation and identity, home environment and family relationships, history of bullying, physical and sexual abuse, and substance use.
    - Explore other types of information would you want to ask an adolescent in the HEADSS exam that would be more specific to LGBT youth. Examples include: To whom/when did you come out? How was this experience? Did it open you up to any unsafe situations? Have you experienced victimization or homo-/transphobia? What is your social situation like in terms of where do you hang out (e.g., bars, clubs)? Some behaviors can lend themselves to unsafe behaviors.

Question 2:

How would you recommend Madison proceed with additional data gathering?

- - - Confidentiality: Recommend that Madison states confidentiality up front, early on & repeated as necessary. Confidentiality usually covers 4 areas (depending on state law): sexual activities, reproductive health (contraceptive use & options counseling), substance use, mental health issues (only if not requiring inpatient hospitalization).
    - Also discuss with Madison about the exceptions to confidentiality. These include harm to self, harm to others, and abuse & neglect.
    - Review the **PEARLS** acronym (Academy of Communication in Healthcare, ACH: <https://www.achonline.org/>), a useful framework for building trust & practicing effective communication, with Madison:
      - **P**artnership: Partner with patients & families through the encounter. Use “we” statements. Align goals.
      - **E**mpathy: Demonstrate empathy both verbally & non-verbally: e.g., I can see this has been difficult for you. Placing a hand gently on Lee’s shoulder may also be helpful.
      - **A**pology: Apologize for misunderstanding or lack of understanding, etc.
      - **R**espect: Listen without judgment. Approach Lee with the intention to help, not to judge.
      - **L**egitimization: Validate Lee’s perspectives and experience.
      - **S**upport: Offer a lifeline and follow up plans.
    - Asking Suicide-Screening Questions Toolkit (ASQ): <https://www.nimh.nih.gov/research/research-conducted-at-nimh/asq-toolkit-materials/emergencydepartment/asq-tool.shtml>
      - Use the 3 simple questions. Follow up with detail if any screens positive.
        - In the past week, have you wished you were dead?
        - In the past few weeks, have you felt that you or your family would be better off if you were dead?
        - In the past week, have you been having thoughts about killing yourself?

Question 3:

What plans would you recommend for Lee for this visit? What follow-up plans?

- - - Assess urgency: Ask Madison to consider “How imminent is Lee’s risk of suicide? How safe is Lee if discharged home at this time?”
    - Take an interdisciplinary approach: Ask Madison to identify interdisciplinary team members: e.g., Adolescent Medicine Specialist, Social Worker, Child & Adolescent Psychiatrist. There is no need to go it alone.
    - Locate local mental health resources.
    - Schedule close follow up.

Question 4:

What resources would you recommend for Madison to learn more?

- See selected materials and resources appendix.

*OPTIONAL Role-Play:*

*Obtain a more detailed history of the adolescent who present with a history of cutting.*

- *Have the group divide into triads: one participant plays the patient (Lee), another plays the faculty, and the 3^rd^ plays the student (Madison)*
- *Play out the scenario using 2-3 of the discussion questions above. You may choose to have the student (Madison) act out the interactions with the faculty as observer, OR have the faculty role-model the interactions with the student as the observer*
- *Use the rest of the time to debrief on how the interactions go in your small group. (There will be an opportunity to share insight with the large group later in the workshop)*

Overall Education Talking Points:

- Focus on your learner learning to ask the “why”; this is especially helpful when using the HEADSS model
- Be aware of your own gaps of knowledge regarding sex and gender issues; acknowledge this with your learner. This can be beneficial in both closing knowledge gaps together and demonstrating life-long learning to your learner.

**References**

1. National Institute of Mental Health. Transforming the understanding and treatment of mental illnesses. <https://www.nimh.nih.gov/research/research-conducted-at-nimh/asq-toolkit-materials/emergencydepartment/asq-tool.shtml>.
2. Academy of Communication in Healthcare. Better Communication. Better Relationships. Better Care. 2020; <https://www.achonline.org/>.
3. Committee On A. Office-based care for lesbian, gay, bisexual, transgender, and questioning youth. Pediatrics. 2013;132(1):198-203.

## Case 2: “I broke my arm!”

*Developed by Eric Kutscher, MD*

Role: Resident

Scenario:

You are a PGY-3 resident working with a clerkship student (M3), Mitch, during his busy ED shift. You assign Mitch to assess John, an 11-year-old transgender boy brought in by his parent for trauma to his right arm. John reports that he has been playing basketball with his friends this afternoon when he tripped and fell on his outstretched arm. His pain is a 10/10 and is localizing to his right shoulder. His past medical and surgical history are notable for a histrelin (Supprelin) implant in his left arm to suppress puberty 1 year ago. He takes no medications, has no allergies, and his vaccines are up to date. On physical exam, John does not note tenderness or signs of trauma along any part of John’s right arm, but appreciates a large hematoma on John’s right shoulder.

In your discussion about John, direct Mitch to focus on how he can ensure a smooth medical evaluation and incorporate his sexual identity into his care.

Discussion Questions:

1. Ask Mitch to explain how the mechanism of trauma does or does not fit with the story provided & the findings on the physical exam. What additional questions would help elicit additional information? What approach would you suggest Mitch take in gathering this information?
2. Mitch asks about the role of histrelin (Supprelin) for puberty suppression, and what Tanner Stage John may be. How would you discuss with Mitch Tanner Stage assessment for John in the ED setting?
3. Based on your suspicion for non-accidental trauma, Mitch suggests that John be admitted for further work up and treatment. Ask Mitch 1) how he would approach roommate assignment for John; 2) if there is a hospital policy in place for transgender patients; 3) how this information should be communicated with John.

## Case 2: “I broke my arm!”

### Instructor Guide with Suggested Responses

Question 1:

In a case where the mechanism of action described doesn’t fit a patient’s injury, further information must be obtained to assess for any non-accidental trauma. Consider the following steps:

- Establish privacy; e.g., Say to the parent, “I routinely give my patients some time to speak with me alone. Could you wait in the waiting area while we chat, and I’ll grab you when we’re finished?”
- Explicitly mention confidentiality (tailor to state law). “As I said, I routinely give all my patients to speak with me alone. Anything you tell me is confidential and I won’t tell your parents or anyone else, unless what we discuss involves you hurting yourself or others. In that case, I would let you know before I tell anyone else. Is there anything you’d like to share with me off the bat?”
- State the facts (objective findings on physical exam) and allow John to respond: e.g., “John, I see you have a large bruise on your shoulder. Can you tell me more about this?”
- Maintain transparency: e.g., “Based on the story you told me, I can’t really explain the large bruise on your shoulder. Most people who fall on their arm would hurt their wrist or elbow, so I’m concerned that we don’t have a good explanation of what happened to you.” If John is hesitant, you may ultimately ask him directly, “Did anyone hurt you intentionally or unintentionally?”
- Ensure supervision and preparation. Make sure a supervisor is present to 1) provide direct supervision; 2) serve as a witness during these conversations. Preparation involves discussing the plan with the Mitch explicitly & in advance. How much Mitch directs the conversations should be based on his demonstrated skillset.

Question 2:

- Encourage intellectual curiosity discuss use of GnRH analogues (often leuprolide (Leupron) or histrelin (Supprelin)) in a precepting setting, reviewing mechanisms of action, indications, and referring students to transgender health specific providers at your institution for more information.
- Tailor to the encounter. GnRH analogues can cause diminished bone mineral density. Closely evaluate for any evidence of fractures on exam, and if found a further discussion about histrelin (Supprelin) use is warranted. Otherwise encourage dietary intake of calcium and have his vitamin D levels monitored closely in the primary care setting. Some providers recommend DEXA scans, but this is not routinely practiced.
- Avoid unnecessary exams. The medical student has explicitly asked about Tanner staging, which can be discussed more in the precepting room. Deferring all genital or chest exams in transgender youth is essential given high rates of reported emotional and physical trauma in the medical setting. It would be inappropriate to perform any sensitive exam components with John.

Question 3:

- Every institution has a different policy regarding rooming for transgender individuals in the hospital. This topic is highly charged and emotional for many patients, as it often feels like a validation or critique of an individual’s trans identity.
- Ensure you know your hospital’s policies and can best explain them to patients and their families.

*OPTIONAL Role-Play:*

*You are the attending physician. You have a medical student with you. You speak to John and his parents.*

- *What else do you want to know?*
- *How will you sensitively and effectively obtain hx about trauma etiology/LGBTQ+*
- *How will you explain the roommate situation to John?*
- *(Can debrief here)*

*John’s parents ask to speak to you alone. They are not happy about how the ED personnel did not view the “sticky note” about preferred pronouns. They do not understand the rooming situation.*

- *They had doubts about histrelin (Supprelin) and want to know if they should have decided against histrelin (Supprelin) for John*
- *You are not fully aware ( knowledge with medication and other content) How do you advocate for care while acknowledging your lack of knowledge (cultural humility, professionalism- uncertainty, )*
- *The student wants to know how to address the patient and asks there any medical-legal ramifications to systems-based errors?*

Overall Education Talking Points:

- How to address one’s own content gaps?
- How to advocate for care while acknowledging your lack of knowledge (cultural humility, professionalism- uncertainty)
  - “I am not that familiar with this, sounds like you are, I would like to hear/learn more at the end of clinic”
- Getting back to learner- closing the loop on the “observer”
- Being aware of your clinic/hospital’s “protocols”
- How to make environment inclusive:
- Aware of how to advocate (EMR, admissions, scheduling etc.)
- Message to staff
- Message to learners

**References**

1. Shumer DE, Nokoff NJ, Spack NP. Advances in the Care of Transgender Children and Adolescents. Adv Pediatr. 2016;63(1):79-102.
2. Olson-Kennedy J, Rosenthal SM, Hastings J, Wesp L. Health considerations for gender non-conforming children and transgender adolescents. 2016; https://transcare.ucsf.edu/guidelines/youth.
3. Mahfouda S, Moore JK, Siafarikas A, Zepf FD, Lin A. Puberty suppression in transgender children and adolescents. The Lancet Diabetes & Endocrinology. 2017;5(10):816-826.
4. Lopez CM, Solomon D, Boulware SD, Christison-Lagay ER. Trends in the use of puberty blockers among transgender children in the United States. J Pediatr Endocrinol Metab. 2018;31(6):665-670.
5. National Center for Transgender Equality and the National Gay and Lesbian Task Force. National Transgender Discrimination Survey Report on health and health care. Washington, D.C.: National Center for Transgender Equality; National Gay and Lesbian Task Force;2010.
6. Vlot MC, Klink DT, den Heijer M, Blankenstein MA, Rotteveel J, Heijboer AC. Effect of pubertal suppression and cross-sex hormone therapy on bone turnover markers and bone mineral apparent density (BMAD) in transgender adolescents. Bone. 2017;95:11-19.

## Case 3: A Question of Risk

*Developed by Adam Wolfe, MD, PhD*

Role: Attending subspecialist physician

Scenario:

Jodie is a 9-year-old transgender girl. Her parents appear to be fully aware and supportive of her plans for gender affirmation. They have been taking Jodie to a local endocrinologist, who plans to administer estrogen therapy as Jodie approaches adolescence. The endocrinologist has referred Jodie to you, the pediatric hematologist, to clear Jodie for estrogen therapy. The family history is remarkable in that Jodie’s biological mother was placed on a combined oral contraceptive medication a few months ago and abruptly developed a pulmonary embolus, for which she is now on long term anticoagulation. Her parents have questions about how to manage any medical risks of doing so.

A primary family member who developed a thrombus at a young age is itself a significant risk factor for a child having increased risk of developing thrombosis at a young age. Jodie is therefore at higher than average risk of developing a thrombotic event if she receives exogenous estrogen therapy, regardless of any other medical tests we might conduct as workup.

Discussion Questions:

As your group evaluates this case, focus on how providers can communicate with patients in this type of situation. Consider the following questions.

1. What would be the best way to share the medical concerns in this case with Jodie and her parents?
2. Jodie’s parents ask you to provide a percent chance that she would have a life-threatening complication from the proposed therapy (there is not an evidence-based numerical answer). How would you counsel them?
3. You have a PGY-3 pediatrics resident working in the hematology clinic with you and she is seeing Jodie with you. How will you teach the resident about best practices to approach the family counseling in this situation?

## Case 3: A Question of Risk

### Instructor Guide with Suggested Responses

Question 1

- Describe generally what provocations make blood clot – vascular injury, stasis of flow, and hypercoagulability - and put estrogen into the context of the latter. Explain that hypercoagulable states are often inheritable.

Question 2

- Transgender women who receive exogenous estrogen therapy without specific other risk factors are considered at very low risk of spontaneous deep venous thrombosis or pulmonary embolism. However, certain formulations of estrogen therapy are associated with greater risks – these are considerations for the family to discuss with the endocrinologist.
- Emphasize that we can only speak in terms of risk factors, not certainties. In this case, it is impossible to know whether Jodie will have a life-threatening clot if she is treated as proposed, just that her risk of an event is higher than other transgender women because of her mother’s history.

Question 3

- Counseling appropriately is the most important part of the encounter. An effective strategy is the same one used for sharing life-altering information, called SPIKES (**S**etting, **P**erception, **I**nvolvement, **K**nowledge, **E**mpathy, **S**ummary). Specifically, in this case:
  - Recognize the patient’s/parent’s perspective and understanding of the situation. Correct any misunderstanding/misinformation.
  - Involve Jodie and her parents in decision making as much as possible.
  - Share clear and understandable medical information. Ask them to “teach back” the information as a show of understanding (instead of just asking “any questions”?).
  - It is crucial to exhibit empathy in this context. Use phrases that exhibit that you understand how important this is to the patient and family. “I can see that this is a very important decision.” Also empathize with how frustrating medical uncertainty can be in this setting. “I wish I could give you a precise answer, but I also want to make you aware of the uncertainty about your risks in this situation.”
- Establish shared goals with the family, typically centered around the health and wellbeing of the patient.
- Depending on the comfort level of the specialists involved, it may not be possible to “clear” Jodie to receive high dose estrogen therapy in the sense that the endocrinologist has requested, given the risks described. At the same time, this may still be a decision the family could make on behalf of the child once they are completely informed of the risks and counseled about symptoms to watch for and other avoidable hypercoagulability risk factors.

OPTIONAL Role-Play:

- In small groups of 3: One participant plays Jodie’s parent; one participant plays the hematologist; one participant is an observer and provides feedback.
- Hematologist: Practice answering Question 1 to Jodie’s parent. Based on the effectiveness of the explanation, the parent should then ask follow- up questions (including Question 2) to probe the certainty of the risk to Jodie if she receives the proposed therapy.
- Also practice effective communication strategies described in the talking points under Question 3 above.

Overall Education Talking Points:

- A subspecialist is often consulted to answer a specific question; it is possible to lose track of the larger ramifications of the answer to a patient and family.
- Explain medical information to families in objective, understandable ways.
- When there is uncertainty or ambiguity as to a medical decision, a family needs to be as informed as possible to make the best decision for a child. When there is greater certainty, it is easier to render a strong recommendation.
- When unwelcome or unexpected information needs to be shared with a patient or family, it is crucial to explore ways to show empathy for the difficult situations/decisions they face.

**References**

1. Arnold JD, Sarkodie EP, Coleman ME, Goldstein DA. Incidence of Venous Thromboembolism in Transgender Women Receiving Oral Estradiol. *J Sex Med.* 2016;13(11):1773-1777.

2. Chan W, Drummond A, Kelly M. Deep vein thrombosis in a transgender woman. *CMAJ.* 2017;189(13):E502-E504.

3. den Heijer M, Bakker A, Gooren L. Long term hormonal treatment for transgender people. *BMJ.* 2017;359:j5027.

4. Wolfe AD, Denniston SF, Baker J, Catrine K, Hoover-Regan M. Bad News Deserves Better Communication: A Customizable Curriculum for Teaching Learners to Share Life-Altering Information in Pediatrics. *MedEdPORTAL.* 2016;12:10438.

## Case 4: 6-year-old walk in

*Developed by Marina Catallozi, MD, MSCE*

Role: Resident

Scenario:

You are precepting the 2^nd^ year outpatient resident (Caroline) for walk-ins. A 6-year-old child assigned female sex at birth and named Mary comes in as a walk-in with distressed parents because the child has been dressing like a boy recently. They are worried their child is going to be bullied at school, so they have been forcing the child to wear dresses and pink bows. They wonder if their child is transgender. Your resident has never had an experience such as this outside of adolescent clinic and is not sure if the child is transgender. She is also not sure if she has the terminology correct.

The resident tells you the parents conveyed that they think this is just a phase, since the mom was a “tomboy” as a child and quickly grew out of it. The father is worried because his family is going to visit because they saw photos of Mary on Facebook and want to discuss a “camp for kids like Mary” they want to pay for this summer.

The resident informs you that she did let the family know she would not sure of the “details” of the terminology. She also informs you that she let them know “it could be JUST a phase; it can be tricky – I will come back with my attending to discuss more.

Discussion Questions:

1. How do you define transgender, gender expansive, and gender dysphoria to your resident? How would you do this in front of the family? To the family?

Consider defining some terms with the family and the resident: this targets teaching on two aspects. It would be still important to follow up with the resident at a later time.

1. How do you inform your family with the resident present about the concept of “it being a phase”?
   1. What words to use? What words to avoid?
   2. Cautioning resident to avoid “just” both here and in any serious situation
2. How do you respond when the family asks, “Is this a phase?” “How do you know?”
3. What do you do if the learner is not comfortable caring for a transgender child?
   1. How do you address this to them?
   2. What else needs to be done?

Case adapted from “Appendix A, Case 4” in: Roth LT, Friedman S, Gordon R, Catallozzi M. Rainbows and “Ready

for Residency”: integrating LGBTQ health into medical education. MedEdPORTAL. 2020;16:11013. Adapted and reproduced under Creative Commons Attribution (CC BY 4.0) license.

## Case 4: 6-year-old walk in

### Instructor Guide with Suggested Responses

Question 1:

- Suggest having such education materials available everywhere: your office, clinics, precepting offices.
- Have definitions readily available.
- Have your resources ready.
- Be knowledgeable as the teaching attending.
- When possible, discuss ahead with your learner.
- Emphasize the importance of not labeling it a “phase.”
- ASK THE CHILD! What name do they want to be called? What pronouns do they want to use?

Question 2:

- Say it - “This is not a phase.” Emphasize the importance of not labeling it a “phase.”
- ASK THE CHILD! What name do they want to be called? What pronouns do they want to use? Bring validity to the child.
- Avoid “just.”

Question 3:

- Say it- “This is not a phase.” Emphasize the importance of not labeling it a “phase.”
- Studies of parents/caregivers of transgender individuals state they first notice signs at an average age of 4 ½ and children on average state they first started feeling different at an average age of 6; however, most do not express or even understand their gender identity until they are teenagers or adults.
- AAP recommends a “gender affirming” approach.
- Focus on what the child says about their gender identity and allow them to determine what forms of gender expression feel comfortable and authentic.
- Social transition can occur at ANY age - adopting gender-affirming hairstyles, clothing, name, gender pronouns, restrooms, and other facilities.

Question 4:

- Discuss with learner: explore their barriers to learning this topic and ask about possible gaps in their knowledge. Remember - they may not have learned this in medical school.
- Provide resources ranging from nomenclature easy-to-read materials to AAP guidelines
- Follow up with them later in the rotation, if possible.
- Work to have more formal learning in the rotation and program.

*OPTIONAL Role-Play:*

- Have the group divide into triads: one will play the Mary’s parent, one will play the faculty, and the 3^rd^ will play the resident (Caroline).
- Use 2-3 of the questions above to play out the scenario. You may choose to have the resident (Caroline) act out the interactions with the faculty as observer, OR have the faculty act out the interactions with the resident as the observer.
- Use the rest of the time to debrief how the interactions go.

Overall Education Talking Points:

1. How to educate both families and learners
2. How to address one’s own bias and best manage a patient/family when there are members of the team whose biases can be harmful
3. How to advocate for care while acknowledging that it can be difficult (cultural humility, professionalism- uncertainty)

- “I understand that this is not something you are very comfortable with but I want to be sure you understand how important gender affirming care can be for individuals and their family and that you can think about how you can be involved in their care.”

1. How to communicate difficult/serious news in a culturally sensitive/culturally humble manner
2. How to model this content domain you as the teacher may not yet feel knowledgeable about
   1. Concept of medical uncertainty
   2. Concept of Cultural Humility
3. Suggest having educational materials available everywhere: your office, clinics, precepting offices (AAP Recommendations):

- Studies of parents/caregivers of transgender individuals state they first notice signs at an average age of 4 ½ and children on average state they first started feeling different at an average age of 6; however, most do not express or even understand their gender identity until they are teenagers or adults.
- The majority of gender expansive children will NOT become transgender adults, but steps to validate a child’s gender identity or expression at this age are reversible
- Many studies have shown that transgender adolescents and adults rarely regret gender transition and the process of transitioning greatly improves their wellbeing
- Conversion or reparative therapy entails trying to change someone’s gender identity and can be incredibly dangerous including electric shocks, hypnosis, institutionalization, or inducing vomiting or paralysis in association with the child’s gender identity.
- The consensus is overwhelmingly against this approach and in many states illegal; however, thousands of teens will still go through conversion
- AAP recommends a “gender affirming” approach
- Focus on what the child says about their gender identity and allow them to determine what forms of gender expression feel comfortable and authentic
- Social transition can occur at ANY age - adopting gender-affirming hairstyles, clothing, name, gender pronouns, restrooms, and other facilities
- ASK THE CHILD! What name do they want to be called? What pronouns do they want to use?
- There are many risks to discouraging or shaming a child’s gender identity/expression; specifically, worsening or prolonging gender dysphoria which can precipitate severe depression, anxiety, self-harm, suicidality
- DO NOT WAIT!
  - - “Watchful waiting”/delayed transition can lead to increased risk of depression, anxiety, school failure, substance abuse, and suicide attempts

**References**

1. Committee on Adolescence. Office-based care for lesbian, gay, bisexual, transgender, and questioning youth. *Pediatrics.* 2013;132(1):198-203.
2. National Institute of Mental Health. Transforming the understanding and treatment of mental illnesses. <https://www.nimh.nih.gov/research/research-conducted-at-nimh/asq-toolkit-materials/emergencydepartment/asq-tool.shtml>.
3. Roth LT, Friedman S, Gordon R, Catallozzi M. Rainbows and “Ready for Residency”: integrating LGBTQ health into medical education. MedEdPORTAL. 2020;16:11013.
