# Supplementary material for: Teaching Sexual Orientation and Gender Identity in Pediatric Clinical Settings: A Training Workshop for Faculty and Residents
Source: MedEdPORTAL. 2021 Apr 5;17:11137. doi: 10.15766/mep_2374-8265.11137 (PMC8034234; doi:10.15766/mep_2374-8265.11137)
Supplement: Supplementary file 1 — Facilitator Guide.docxPatient Vignettes.pptxDidactic Presentation.pptxSelected Educational Resources.docxCase Discussion with Role-Play Opportunities.docxEvaluation Form.docx [file mep_2374-8265.11137-s001.zip › F. Evaluation Form.docx]

# Appendix F

Workshop Evaluation Form

**Title of Workshop: Teaching Sexual Orientation and Gender Identity in Pediatric Clinical Settings: A Training Workshop for Faculty and Resident**

**Date:**

**Speaker(s):**

Learning objectives

At the end of this workshop, participants should be able to:

1. Define key concepts of sexual orientation and gender identity as related to pediatric clinical care.
2. Apply these concepts to commonly encountered clinical scenarios related to sexual orientation and gender identity.
3. Identify challenges and opportunities for teaching sexual orientation and gender identity in routine clinical educational duties in the general, subspecialty, and acute care pediatric settings.

| **Satisfaction, Format and Value** | Strongly Disagree  1 | Disagree  2 | 3 | Agree  4 | Strongly  Agree  5 |
| --- | --- | --- | --- | --- | --- |
| Overall, the workshop was effective |  |  |  |  |  |
| Overall, the speakers were effective |  |  |  |  |  |
| The format of this activity was appropriate for its content |  |  |  |  |  |
| This activity was a worthwhile investment in my professional development |  |  |  |  |  |

| **Objectives and Learning** | Strongly Disagree  1 | Disagree  2 | 3 | Agree  4 | Strongly  Agree  5 |
| --- | --- | --- | --- | --- | --- |
| I learned new knowledge and skills from this activity |  |  |  |  |  |
| I will apply the knowledge and skills |  |  |  |  |  |
| This activity is relevant to my professional role |  |  |  |  |  |

How can we improve the activity to make it more relevant?

Thinking about the knowledge and skills you learned at this conference, please describe the **ONE** most important thing you plan to apply once you return to your home institution.

Please describe your teaching background regarding this content and how you will incorporate learning from this workshop into your teaching activities. (This feedback is very helpful for future endeavors-thank you)

Was the content appropriate for your learning and application process? Should it have included more or less learning principles?

**Comments:**
